# Multidisciplinary 3D geological-petrophysical reservoir characterization of Abu Sennan Field, Abu Gharadig Basin, Egypt

**DOI:** 10.1038/s41598-025-13462-w

**Published:** 2025-08-07

**Authors:** Mohamed Mamdouh, Adel Ali Ali Othman, Taher Mostafa

**Affiliations:** Geology Department, Faculty of Science, Al-Azhar University, P.O. Box 11884, Nasr City, Cairo, Egypt

**Keywords:** Seismic interpretation, Petrophysical analysis, Petrographical analysis, Reservoir modeling, Abu Sennan Field, Western Desert, Geology, Geophysics

## Abstract

Reservoir heterogeneity within the Cretaceous Abu Roash and Bahariya formations of the Abu Sennan Field (Western Desert, Egypt) presents a significant challenge to hydrocarbon prospect evaluation. This study applies an integrated approach combining well logs from four wells, core analysis, and 2D seismic data to assess reservoir quality and structural framework. The workflow includes: (1) petrophysical evaluation to quantify shale volume (Vsh), effective porosity (ϕ_e_), and hydrocarbon saturation (S_h_) across five reservoir intervals—Abu Roash C (net pay 1–32.5 m, Vsh 29–35%, ϕ_e_ 19–29%, S_h_ 52–67%), Abu Roash D (7–10.5 m, Vsh 2–13%, ϕ_e_ 17–23%, S_h_ 60–90%), Abu Roash E (3.4–48.6 m, Vsh 20–30%, ϕ_e_ 18–24%, S_h_ 62–76%), Abu Roash G (3–12.5 m, Vsh 11–18%, ϕ_e_ 22–24%, S_h_ 58–73%), and Bahariya (2.5–52.5 m, Vsh 17–27%, ϕ_e_ 15–26%, S_h_ 46–77%); (2) seismic interpretation identifying a NW–SE-trending horst structure bounded by E–W and NNW–SSE fault systems, which compartmentalize the reservoirs; and (3) 3D static modeling to visualize the distribution of facies, porosity, and saturation. The results highlight the AR-D-01 structural closure within the Abu Roash D member as a high-potential prospect, featuring dissolution-enhanced vuggy porosity (ϕ_e_ 17–24%) and elevated hydrocarbon saturation (Sₕ 60–90%) corroborated by depth structure maps and petrophysical property models. Overall, the study demonstrates that fault-controlled diagenesis improves reservoir quality in grainstone facies, offering a reliable framework for targeted hydrocarbon exploration in heterogeneous systems.

## Introduction

**Fig. 1:**
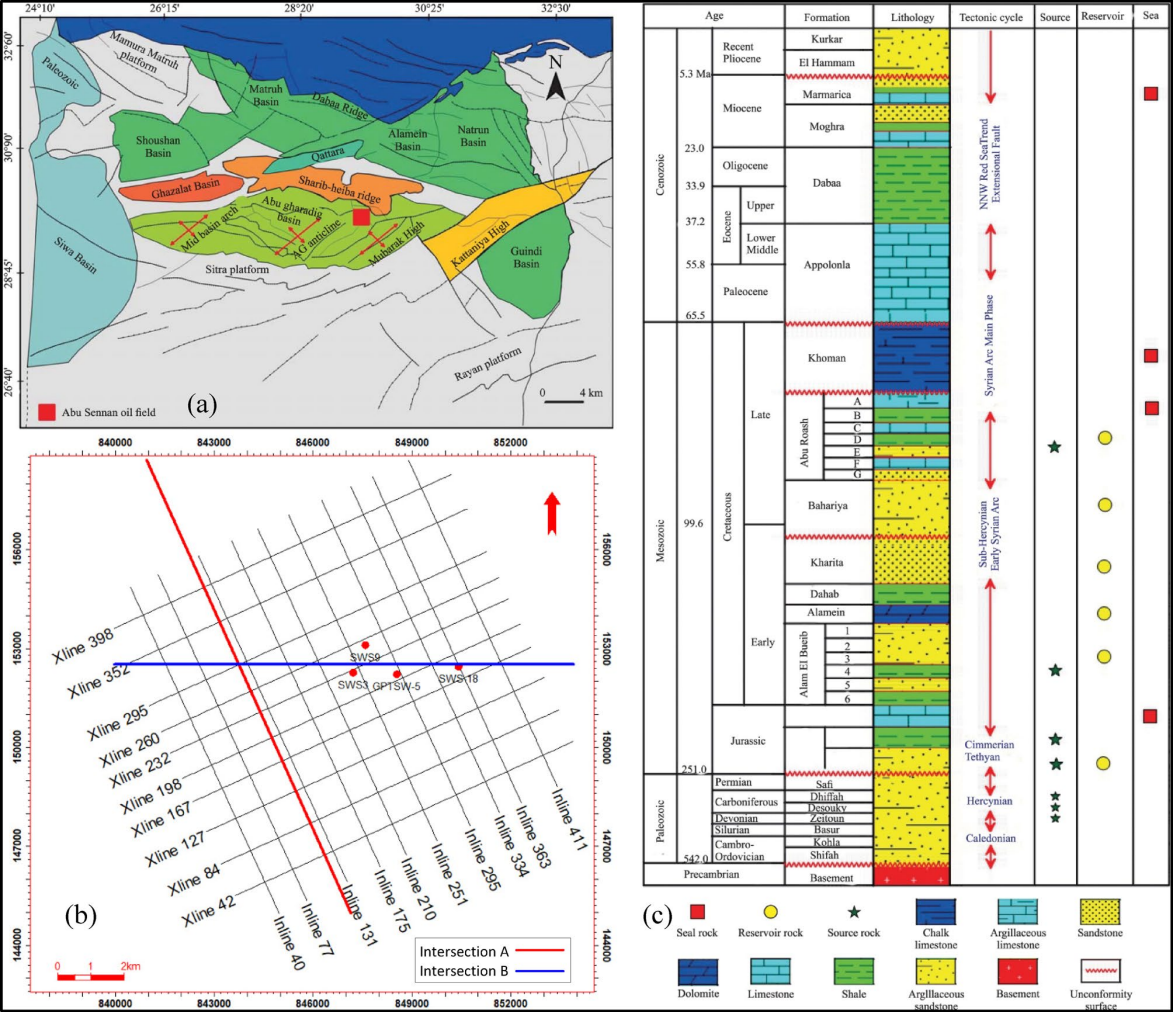
(**a**) Regional location map of the study area. (**b**) Distribution map of seismic data, well locations, and cross-section lines A and B. (**c**) Composite lithostratigraphic column for the study area (modified after^14^).

The Abu Sennan Field, located in the Western Desert of Egypt, represents a significant hydrocarbon province with complex geological and petrophysical characteristics. This field is part of the prolific Western Desert Basin, which has been a focus of extensive exploration and production activities due to its rich petroleum potential^1–3^. The field’s reservoir rocks, primarily within the Bahariya and Abu Roash formations, exhibit substantial heterogeneity in lithology, porosity, and permeability, necessitating advanced geological and petrophysical modeling for effective reservoir characterization and management. Through extensive and prolonged geological investigations, the Western Desert of Egypt has emerged as a region of significant hydrocarbon potential, characterized by the identification of key reservoir and source rock formations. Notably, the Upper Cretaceous Bahariya Formation and the Middle Jurassic Khatatba Formation have been recognized as critical contributors to the area’s petroleum systems^4–6^. The Abu Gharadig Basin, located in the northern part of the Western Desert, is recognized as a primary hydrocarbon-producing basin. This is attributed to the substantial thickness of its sedimentary succession, which includes prominent and extensive primary reservoirs. Additionally, the results of comprehensive source rock analyses confirm the basin’s effectiveness in hydrocarbon generation and expulsion, further supporting its significant contribution to petroleum production within the region^7–9^. The Abu Sennan oil field is situated in the eastern part of the Qattara Depression within the Abu Gharadig Basin, encompassing an area of approximately 300 km^2^. Geographically, it lies between latitudes 29°30′N and 29°40′N and longitudes 28°30′E and 28°40′E (Fig. 1), approximately 200 km west of the Nile River and 20 km south of the Abu Gharadig Gas Field^10–12^. The Abu Roash and Bahariya formations within the field exhibit heterogeneous lithological compositions, accompanied by significant lateral and vertical variations in reservoir parameters, underscoring their complexity and significance in hydrocarbon exploration^13,14^. The Abu Gharadig Basin, including the Abu Sennan Field, has been extensively studied from diverse geological and geophysical perspectives^15–19^. Detailed investigations of the petroleum system elements and processes in the Abu Sennan area have established the Bahariya Formation as a primary reservoir with substantial hydrocarbon potential^14,20^. While previous research predominantly focused on the Bahariya Formation as a reservoir rock, the novelty of this study lies in its dual focus, examining both the Abu Roash Member and Bahariya reservoirs.

The primary objective of this research is to integrate all available subsurface geological and petrophysical data to construct 3D static reservoir models for the Abu Roash and Bahariya reservoirs. Moreover, the study seeks to identify new prospects for field development, offering insights to optimize production rates and provide a comprehensive framework for developing the Abu Sennan oil field. By combining advanced geological and petrophysical modeling techniques, this research aims to enhance the understanding of the reservoir heterogeneity and contribute to the efficient management of hydrocarbon resources in the region.

To contextualize this analysis, the geological framework of the Abu Gharadig Basin—including stratigraphic architecture and tectonic evolution—which fundamentally controls reservoir distribution and compartmentalization is detailed in the following section.

## Geological setting

The Abu Gharadig Basin, extending from the Precambrian to Quaternary^21^, hosts a lithostratigraphic architecture that mirrors the northern Western Desert, as exemplified by the Abu Sennan oil field (Fig. 1a ^14^)^22^. The stratigraphic succession begins with the Lower Jurassic Wadi El-Natrun Formation, dominated by sandstone and shale, transitioning conformably into the Middle Jurassic Khatatba Formation—a heterogeneous sequence of sandstone, shale, and limestone. The Khatatba is overlain by the Lower Cretaceous Masajid Formation, characterized by limestone-shale deposits, which is unconformably succeeded by the Alam El-Bueib Formation (limestone and sandstone). Subsequent unconformities mark the transition to the Lower Cretaceous Alamine Dolomite Formation, followed conformably by the Dahab Shale and Kharita Formations (sandstone-shale assemblages). The Upper Cretaceous Bahariya Formation, a sandstone unit interbedded with shale and limestone^23–25^, unconformably overlies the Kharita Formation and grades conformably into the carbonate-rich Abu Roash Formation. The Bahariya is subdivided into seven members (A–G), reflecting cyclical shifts between limestone, sandstone, and shale. The Abu Roash, in turn, is unconformably capped by the Upper Cretaceous Khoman Formation (chalk and shale). Post-Cretaceous sedimentation includes the Paleocene–Eocene Apollonia Formation (limestone-shale), conformably overlain by the Middle Eocene–Oligocene Dabaa Formation (predominantly shale) and succeeded by the Miocene Moghra Formation (mixed siliciclastic-carbonate facies) (Fig. 1c ^14^).

### Tectonostratigraphic evolution

The basin’s structural framework originated during the Jurassic as an east–west–trending intracratonic half-graben, shaped by Tethyan rifting, followed by Cretaceous subsidence^26^. In the late Cretaceous period, the structure of the basin changed due to the Syrian Arc Deformation, creating new fault lines that run from northeast to southwest. These events had a major impact on oil and gas systems, with the Bahariya and Abu Roash formations being the main sources of oil in Abu Sennan.

### Regional tectonic context

Three main tectonic phases shaped northern Egypt: (1) Paleozoic–Triassic trends that go from northwest to west-northwest, (2) Cretaceous trends from the east-northeast linked to the Syrian Arc, and (3) Late Eocene–Oligocene trends that go northwest (toward the Gulf of Suez) and north-northeast (toward Aqaba). Normal fault systems, which can be seen in seismic and well data^27^, control the underground structure of the northern Western Desert, showing a pattern of highs and lows that run from north-northwest to south-southeast. The Abu Sennan area had normal faults running from northeast to southwest during the Early Jurassic to Cretaceous periods, which were later turned upside down in the Late Cretaceous to Middle Eocene, creating anticlinal traps. Tertiary deformation further imposed northwest–southeast trends, extending from Siwa to the Nile Delta^28^.

This tectonic-stratigraphic framework directly informs our integrated methodology (“Data and Methodology” section), where seismic interpretation resolves fault-bounded structures, and well-log analysis targets the heterogeneous Abu Roash/Bahariya reservoirs described above.

## Data and methodology

This research employs a comprehensive dataset that includes complete well records from four wells intersecting reservoir intervals in the studied area, in addition to core data from one well, which features thin section analyses. A 2D seismic survey covering the entire Abu Sennan field (Fig. 1b) was included to aid in reservoir evaluation, structural framework modeling, and the development of three-dimensional static facies and property models. The subsequent sections systematically outline thdataset andnd the methodologies employed.

### Well logging data

This investigation utilizes a curated dataset derived from four strategically selected wells within the study region, each containing a comprehensive suite of geophysical well logs (gamma-ray, resistivity, neutron, density, and sonic) archived in LAS and ASCII formats. These high-resolution datasets enabled a multidisciplinary analytical framework, combining qualitative lithological interpretation with quantitative petrophysical evaluations. The integrated methodology supported precise delineation and characterization of hydrocarbon reservoirs, with particular emphasis on the stratigraphically complex Abu Roash and Bahariya formations.

A suite of petrophysical parameters underpins our formation evaluation: volume of shale (Vsh), total and effective porosity (ϕt, ϕe), water saturation (Sw), hydrocarbon saturation (Sh), formation resistivity (Rt), and net-to-gross pay (N/G). These parameters quantify clay content, pore-space available for fluids, and fluid distribution. Well-log analysis combines lithology and porosity logs, each sensitive to different rock and fluid properties. Gamma-ray and spontaneous‐potential curves—high in shaly intervals and low in clean sands or carbonates—delineate clay-rich versus reservoir facies. Density logs infer bulk density (ρb) by measuring electron density; porosity follows once matrix and fluid densities are specified. Neutron logs track hydrogen concentration to estimate fluid-filled porosity; a crossover (density-low with neutron-low) often flags gas^12,29–31^.

#### Lithological identification

The lithological composition of the examined formations was established through the analysis of data obtained from multiple geophysical logging tools, including gamma-ray, resistivity, density, and neutron logs. Lithological classification was further refined through the application of graphical interpretation methods, including M–N and neutron-density cross plots. The M–N cross plot is a robust petrophysical tool developed to differentiate lithologies and evaluate mineralogy in subsurface formations by integrating neutron, density, and sonic log measurements. Consequently, cross-plotting these parameters enables a systematic classification of lithological units^32,33^. This method, first introduced by^34^, leverages the contrasting responses of these porosity logs to matrix minerals and pore fluids. The M and N parameters are calculated as follows:

1 $$N=\frac{\left({\phi }_{Nfl}-{\phi }_{Nlog}\right)}{\left({\rho }_{b}-{\rho }_{f}\right)}$$

2 $$M=\frac{\left(\Delta {t}_{{f}_{l}}-\Delta {t}_{log}\right)}{{(\rho }_{b}-{\rho }_{f})}*0.01$$

where Φ_Nfl_ is fluid neutron porosity, Φ_Nlog_ is neutron porosity, ρb​ is bulk density, ρ_fluid_​ is fluid density, Δt_fl_​ is the fluid transit time, and Δt_log_​ is the sonic transit time^35^. The normalization of fluid properties reduces the influence of pore fluids, allowing the plot to emphasize matrix lithology.

This cross plot is particularly effective in distinguishing between common sedimentary minerals such as quartz, calcite, dolomite, and evaporites (e.g., anhydrite), which occupy distinct regions on the M–N plane due to their unique neutron-density-sonic responses^36^. For example, dolomite exhibits higher M values due to its elevated sonic transit time relative to limestone, while quartz’s lower neutron porosity shifts its N value.

The other graph used for lithological identification in this study is the neutron-density cross plot. The neutron-density cross plot is a foundational petrophysical tool used to discriminate lithologies and characterize subsurface formations by integrating neutron porosity and bulk density log measurements. Neutron logs respond primarily to hydrogen concentration, which correlates with porosity and the presence of hydrogen-rich fluids (e.g., water, hydrocarbons) or clay-bound water. Density logs, conversely, measure electron density, which is inversely proportional to bulk density and influenced by both matrix mineralogy and pore fluids. When plotted against one another, these measurements create distinct clusters or trends corresponding to specific lithologies, enabling geoscientists to differentiate quartz-rich sandstones, carbonates (limestone, dolomite), evaporites (e.g., anhydrite), and clay-rich shales^37^.

##### Shale volume calculation

Shale volume (Vsh) is a critical parameter in petrophysical evaluations, as it affects porosity, permeability, and hydrocarbon saturation calculations. Shale content impacts reservoir quality by reducing effective porosity and increasing water saturation due to its conductive properties. Several methods exist to estimate Vsh, including gamma-ray (GR) logs and neutron-density cross-plots. In this work, both gamma-ray logs and neutron-density cross-plots were utilized for shale volume estimation. The gamma-ray log is the most widely used tool for shale volume estimation due to the high radioactive response of shale compared to clean formations. The GR-based shale volume ($\text{VshGR})$ is calculated as:

3 $${V}_{shGR}=\frac{G{R}_{\text{log}-G{R}_{min}}}{G{R}_{\text{max}-G{R}_{min}}}$$

A more robust approach combines neutron and density porosity logs, where deviations from the clean matrix line indicate shale content. The neutron-density shale volume (VshND) is derived as

4 $${V}_{sh\left(ND\right)}=\frac{{\phi }_{N}-{\phi }_{D}}{{\phi }_{Nsh}-{\phi }_{Dsh}}$$

The definitive shale volume was taken as the smaller of the two log-derived estimates—Vsh(GR) and Vsh(ND) to minimize overestimation of clay content, which could adversely impact porosity and permeability calculations^38^.

##### Porosity and fluid saturation

The comprehensive suite of well-logging data, including neutron and density logs, facilitated robust estimation of both total and effective porosity through neutron-density crossplot analysis. Shale volume (Vsh), previously derived from gamma-ray log and neutron-density cross-plot interpretation, was employed to correct total porosity for clay-bound water effects, yielding effective porosity (фₑ) pertinent to hydrocarbon storage capacity. The neutron, density, total, and effective porosity are calculated using the following equations^39–43^:

5 $${\text{Density porosity}}\left( {\phi_{{\text{D}}} } \right): \, \phi_{{\text{D}}} = \, \left( {\rho_{{{\text{ma}}}} {-} \, \rho_{{\text{b}}} } \right) \, / \, \left( {\rho_{{{\text{ma}}}} {-} \, \rho_{{\text{f}}} } \right)$$

6 $${\text{Neutron porosity}}\left( {\phi_{{\text{N}}} } \right): \, \phi_{{\text{N}}} = \, \left( {\phi_{{{\text{Nlog}}}} {-} \, \phi_{{{\text{Nma}}}} } \right) \, / \, \left( {\phi_{{{\text{Nfl}}}} {-} \, \phi_{{{\text{Nma}}}} } \right)$$

7 $${\text{Total porosity}}\left( {\phi_{{\text{T}}} } \right): \, \phi_{{\text{T}}} = \, \left( {\phi_{{\text{D}}} + \, \phi_{{\text{N}}} } \right) \, /{ 2}$$

8 $${\text{Effective porosity}}\left( {\phi_{{\text{e}}} } \right): \, \left( {\phi_{{\text{e}}} } \right) \, = \, \phi_{{\text{t}}} \, \cdot \, \left( {{1 }{-}{\text{ V}}_{{{\text{sh}}}} } \right)$$

where: ρ_ma_ = matrix density, ρ_b_ = bulk (measured) density, ρ_f_ = fluid density, ϕ_Nlog_ = raw neutron-porosity reading, ϕ_Nma_ = neutron porosity of the matrix, ϕ_Nfl_ = neutron porosity of the formation fluid, V_sh_ = shale volume.

Formation water resistivity (Rw) was determined using Pickett plot methodology, wherein deep resistivity (Rt) was plotted against porosity on logarithmic scales. This approach enabled the derivation of Rw from the y-intercept of the clean wet-trend line, providing a critical input for saturation modeling^44^. Given the shaly sandstone lithology of the investigated reservoirs, the dual-water model (DWM) was selected for water saturation (Sw) calculations. This model addresses the distinct conductive contributions of two pore-water phases: (1) free formation water in the interconnected pore network and (2) bound water associated with clay mineral surfaces^45^. The DWM fundamentally modifies the Waxman-Smits formulation by explicitly accounting for 1. Clay-bound water conductivity: Arising from counterions in the electrical double layer of clay minerals, 2. Free-water conductivity: Governed by bulk electrolyte properties in the central pore spaces.

The model operates under the key assumption that both conduction pathways share equivalent geometric distributions—a simplification that has proven effective in moderately shaly formations^46,47^. The governing equation is expressed as

9 $$\frac{1}{{R}_{t}}=\frac{{S}_{w}^{n}}{F}* \left[\frac{1}{{R}_{w}}+\frac{{V}_{Q}* {Q}_{v}}{{S}_{wt}}* \left(\frac{1}{{R}_{cw}}-\frac{1}{{R}_{w}}\right)\right]$$

where:

- *R*_*t*_ = Formation resistivity (Ω·m)
- *R*_*w*_ = Formation water resistivity (Ω·m)
- *R*_*cw*_ = Bound water resistivity (Ω·m)
- *Q*_*v*_ = Effective concentration of clay counterions (meq/mL)
- *V*_*Q*_ = Clay-associated water volume (fractional)
- *F* = Formation resistivity factor (dimensionless)
- n = Saturation exponent (typically ≈ 2)

Following S_w_ determination, the hydrocarbon saturation (S_hr_) was derived as the complementary fraction of pore space:

10 $${S}_{hr}= 1 - {S}_{w}$$

### Petrography

A comprehensive petrographic study was conducted to characterize the Abu Roash “D” member (AR“D” m.) reservoir, utilizing 10 thin sections rigorously prepared from conventional and side-wall core plugs extracted from a representative well (SWS-3 well) (Figs. 8, 9). The thin-section preparation and analytical workflow were executed. Petrographic analysis was performed using polarized-light microscopy to quantify mineralogical composition, texture, and porosity. Lithological compositions derived from thin-section analysis exhibited strong concordance with wireline log interpretations, confirming the reliability of petrographic data^48^. This synergy validates the depositional and diagenetic models inferred from core-log integration, bridging microscale petrographic observations with macroscale reservoir properties.

### Seismic interpretation

A comprehensive 2D seismic survey was acquired and interpreted to delineate the subsurface structural framework of the study area. The interpretation workflow began with the critical seismic-well tie procedure, which was significantly enhanced by the availability of multiple check-shot surveys^19,31,49^. These surveys provided robust time-depth relationships, enabling precise correlation between well log data and seismic reflections^13,50^.

The seismic-well tie served as the foundation for identifying and mapping key stratigraphic surfaces throughout the study area^51^. Following this calibration, we systematically interpreted the target horizons, which facilitated the generation of detailed structure contour maps. These maps revealed promising structural prospects for further evaluation.

The computer-assisted seismic interpretation methodology focused on.

- Structural framework analysis
- Formation mapping of the Abu Roash and Bahariya reservoirs
- Hydrocarbon potential assessment

The seismic interpretation workflow progressed through several key stages:

- Initial seismic-well tie establishment using check-shot data
- 3D horizon tracking of target formations
- Structural interpretation, including fault identification
- Generation of time-structure maps for interpreted horizons
- Time-depth conversion using check-shot derived velocity models
- Creation of depth-structure maps for reservoir evaluation

Fault interpretation revealed three predominant fault trends affecting the study area. The time-depth conversion process, calibrated with check-shot data, ensured accurate transformation of time-domain interpretations to depth-domain structural models. This integrated approach provided a robust geological framework for reservoir characterization and prospect evaluation.

### Reservoir modeling

The construction of 3D structural, facies, and petrophysical property models constitutes the fundamental methodology for achieving this study’s objectives. Integration of seismic and petrophysical datasets formed the critical foundation for facies and property modeling, as these data provide essential constraints on lithotype distributions and rock property heterogeneity. Static reservoir modeling, a cornerstone of reservoir characterization, was employed to delineate reservoir architecture and quantify spatial variations in key parameters^14,19^.

The workflow initiated with facies modeling, utilizing well-derived lithology logs (e.g., gamma-ray, neutron-density) from all wells penetrating the Abu Roash and Bahariya reservoir intervals. A geostatistical approach (e.g., sequential indicator simulation) was implemented to honor vertical and lateral facies trends observed in core and log data. Subsequently, petrophysical modeling was performed through co-kriging techniques^13,49,52^.

#### Structural modeling

The development of a reliable 3D structural model necessitates systematic execution of critical geological and geostatistical workflows. This study implemented a hierarchical approach comprising four key processes: (1) fault modeling, (2) pillar-gridding, (3) horizon construction, and (4) stratigraphic layering. Each step was designed to preserve geological integrity while ensuring computational efficiency for subsequent reservoir simulations^14,31^.

##### Fault modeling and pillar-gridding

Fault framework construction began with pillar-gridding, a technique that generates a 3D cellular lattice aligned with interpreted fault surfaces^53^. This method:

- Creates a deformable structural skeleton honoring fault geometry
- Orients grid cells parallel to fault planes to minimize discretization artifacts
- Enables seamless integration with flow simulation grids

The resulting pillar grid serves as the topological foundation for both static geological modeling and dynamic reservoir simulation.

##### Horizon construction

Interpreted seismic horizons were calibrated to well tops and were converted into continuous surfaces using kriging interpolation. These surfaces define major stratigraphic boundaries (e.g., sequence boundaries, flooding surfaces) and constrain vertical property distribution in the 3D grid^54–56^.

##### Stratigraphic layering

To resolve vertical heterogeneity, each horizon-bounded zone was subdivided into sublayers using proportional layering^57^. This process preserves depositional thickness trends observed in core/log data, maintains cell aspect ratios suitable for numerical simulation, and enables high-resolution property population (porosity, permeability).

#### Facies and petrophysical modeling

The integration of log-derived facies classification and geostatistical property modeling formed the methodological core of this reservoir characterization study. A deterministic clustering algorithm, applied to multi-log responses (gamma-ray, density-neutron, resistivity), generated discrete facies logs across the stratigraphic intervals studied. These logs were subsequently upscaled to the seismic grid resolution using arithmetic averaging weighted by vertical heterogeneity, then spatially distributed through Sequential Indicator Simulation (SIS) to honor well data and facies proportion curves^57^. Facies architecture critically constrained petrophysical property modeling, as lithotype distributions govern porosity/permeability relationships^58^**.**

Collectively, these methods generate the key results presented in “Results” section: (1) petrophysical properties from well-log analysis, (2) microfacies/diagenetic insights from petrography, (3) fault geometries from seismic interpretation, and (4) 3D reservoir models integrating these datasets.

## Results

### Well Logs Interpretation

**Fig. 2:**
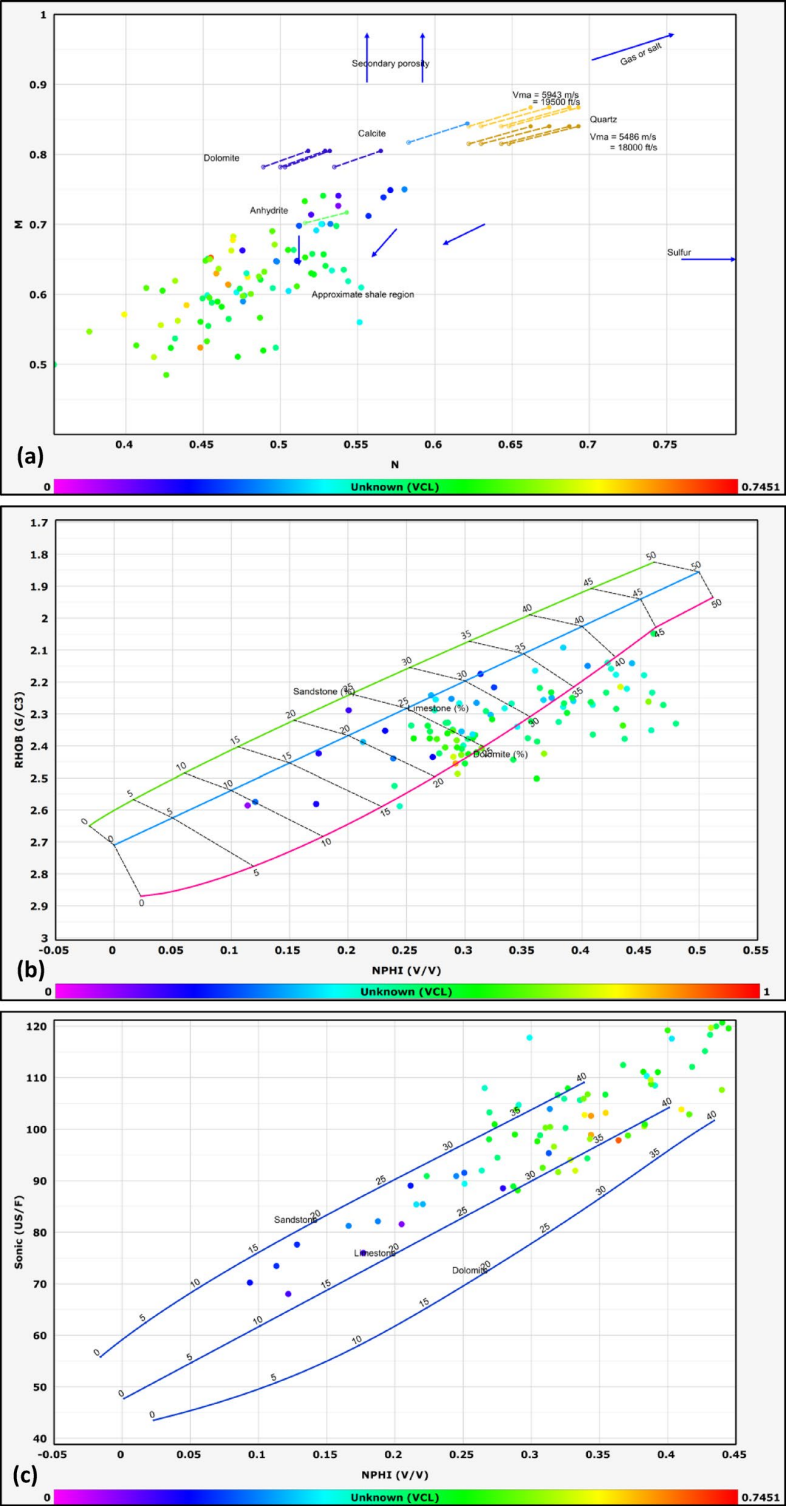
Lithological discrimination cross-plots for the Abu Roash C Member: (**a**) Neutron-Matrix (NM) cross-plot, (**b**) Neutron-Density cross-plot, (**c**) Neutron-Sonic cross-plot.

**Fig. 3:**
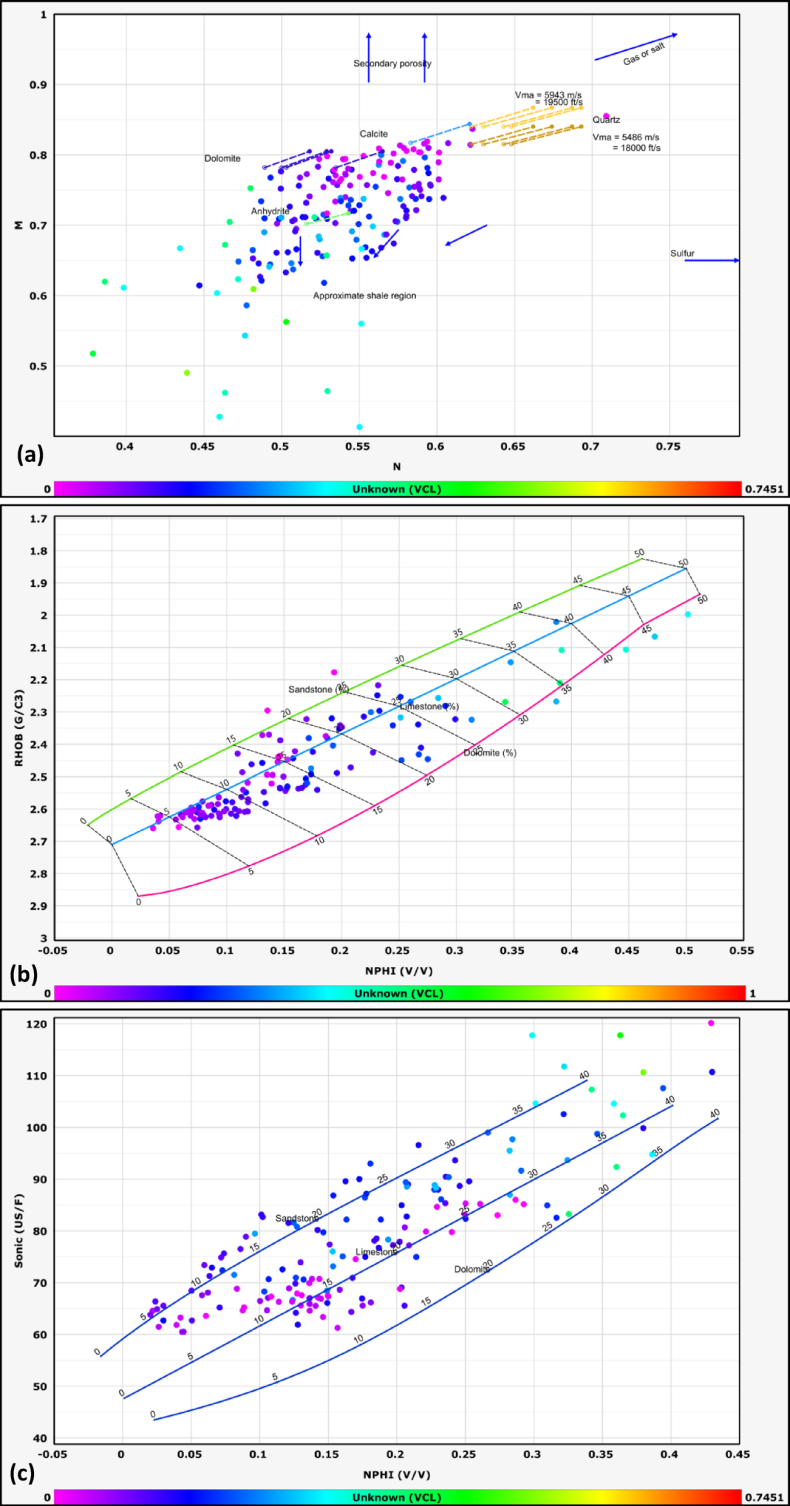
Lithological discrimination cross-plots for the Abu Roash D Member: (**a**) Neutron-Matrix (NM) cross-plot, (**b**) Neutron-Density cross-plot, (**c**) Neutron-Sonic cross-plot.

**Fig. 4:**
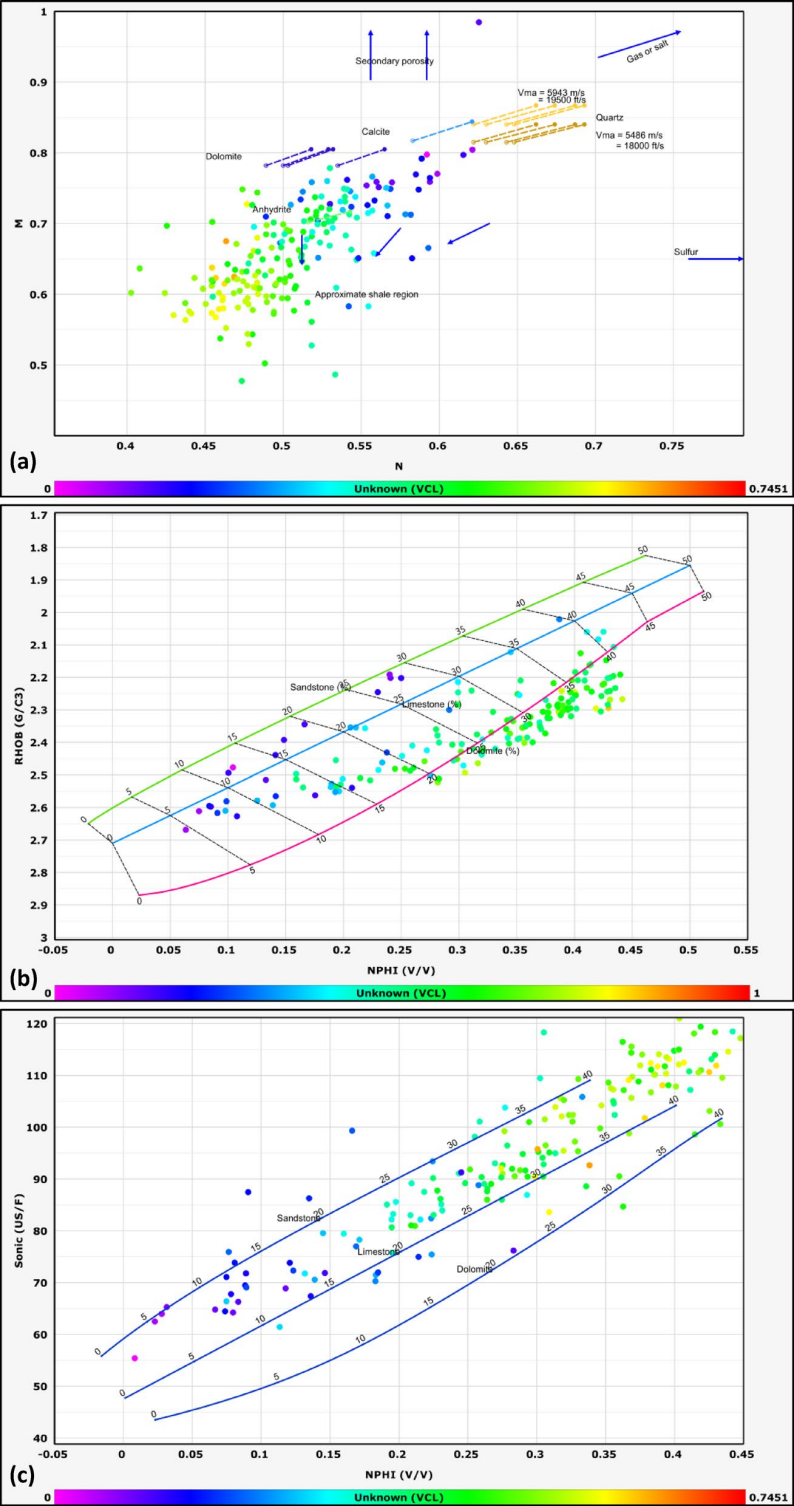
Lithological discrimination cross-plots for the Abu Roash E Member: (**a**) Neutron-Matrix (NM) cross-plot, (**b**) Neutron-Density cross-plot, (**c**) Neutron-Sonic cross-plot.

**Fig. 5:**
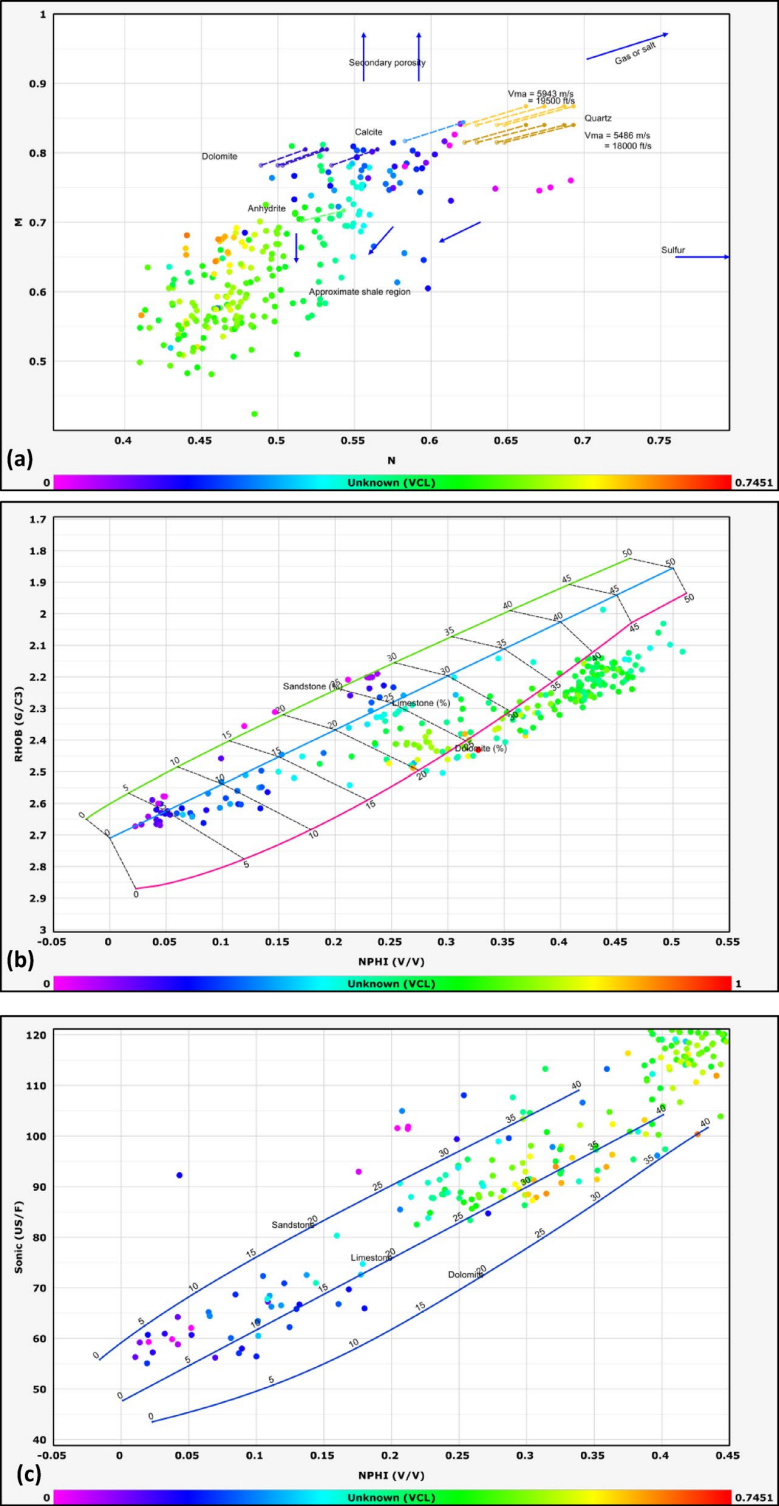
Lithological discrimination cross-plots for the Abu Roash G Member: (**a**) Neutron-Matrix (NM) cross-plot, (**b**) Neutron-Density cross-plot, (**c**) Neutron-Sonic cross-plot.

**Fig. 6:**
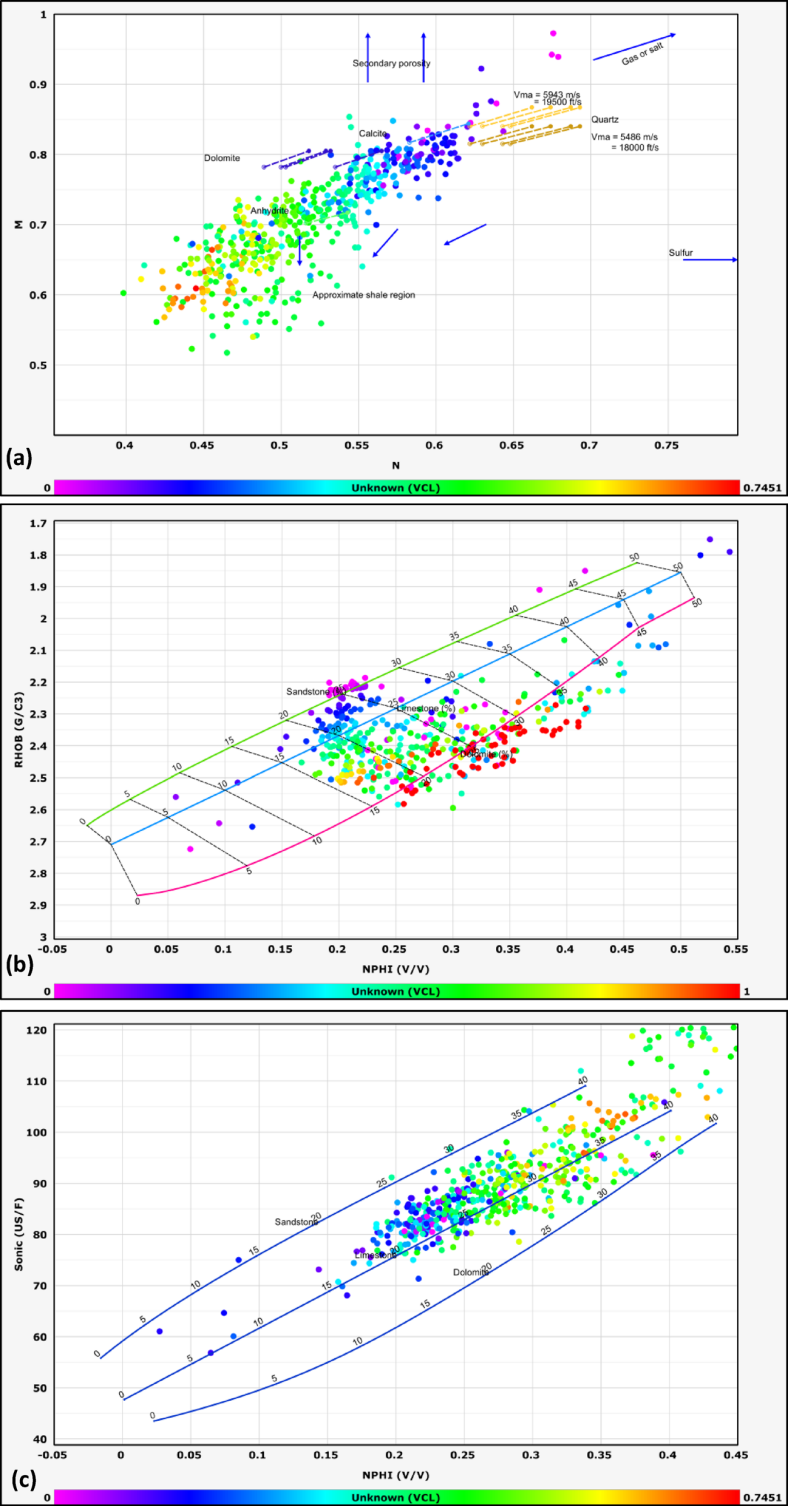
Lithological discrimination cross-plots for the Bahariya Formation: (**a**) Neutron-Matrix (NM) cross-plot, (**b**) Neutron-Density cross-plot, (**c**) Neutron-Sonic cross-plot.

**Fig. 7:**
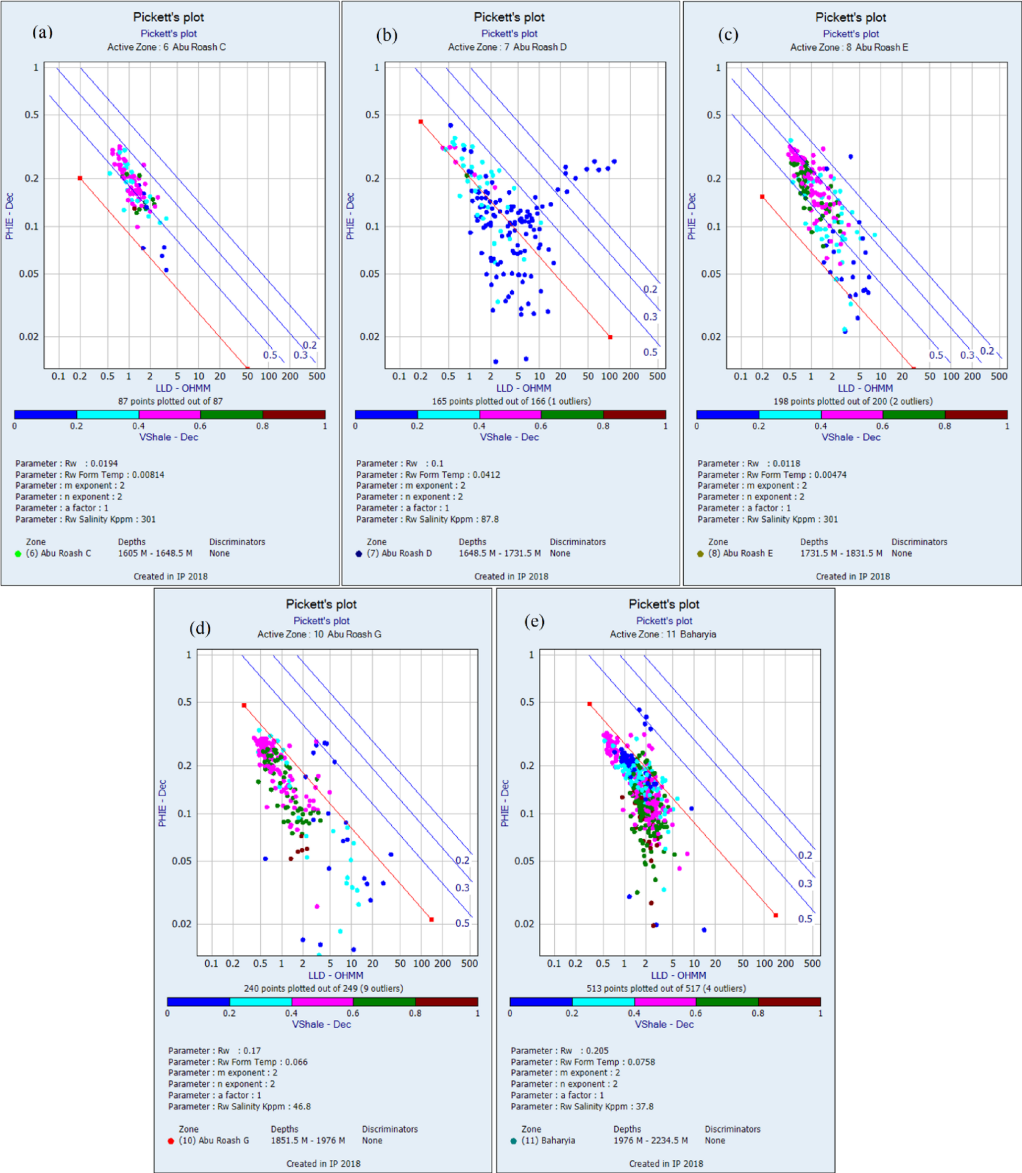
Pickett plot analysis for formation water resistivity (Rw) determination: (**a**) AR/C Member, (**b**) AR/D Member, (**c**) AR/E Member, (**d**) AR/G Member, and (**e**) Baharyia Formation.

**Fig. 8:**
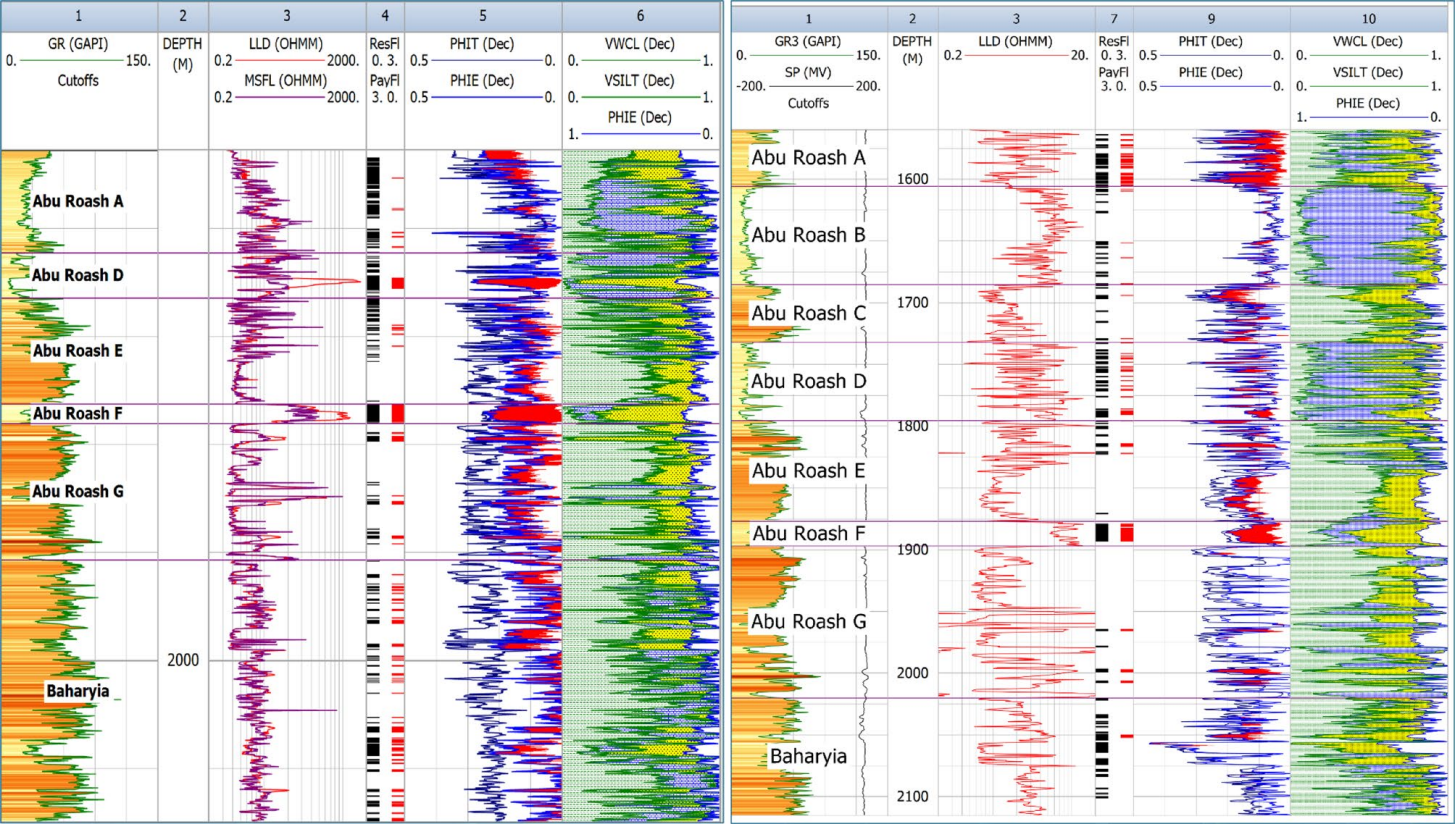
Lithosaturation plots for representative wells, displaying formation lithology and vertical distribution of key petrophysical parameters within the reservoir intervals.

The lithology of the formations studied in the area was identified using logs and log-derived cross-plots. NM, neutron-density, and neutron-sonic cross-plots were used (a, b, and c, respectively, in Figs. 2, 3, 4, 5, and 6, and the lithological compositions of the studied formations were revealed. Formation water resistivity for each reservoir interval was determined using Pickett plot analysis (Fig. 7). A computer-processed interpretation was conducted on the potential reservoir layers in the area under investigation, and the lithosaturation panels were visualized. Figure 8 shows vertically the distribution of the petrophysical parameters and the lithosaturation (lithological composition, effective porosity, and fluid saturations). The resulting logs were used to build facies and petrophysical models for the Abu Roash Members and Bahariya Formation. GR, neutron, and density logs were used to calculate the shale content in the layers. The shale content (Vsh) varied between 29 and 35% for the Abu Roash C member, 2% and 13% for Abu Roash D, 20% and 30% for Abu Roash E, 9% and 23% for Abu Roash F, 11% and 18% for Abu Roash G, and 16% and 44% in the Bahariya Formation. Neutron, density, and sonic logs were used to calculate the effective porosity used with the resistivity logs to identify the fluids and estimate their saturations. The Abu Roash C member has effective porosity values from 19 to 29%, and Abu Roash D has values between 17 and 23%. The effective porosity values for Abu Roash E, F, and G and the Bahariya Formation are 18–24%, 16–20%, 22–24%, and 15–26%, respectively. The estimated oil saturation values for the studied formations are 52–67%, 60–90%, 62–76%, 61–90%, 58–73%, and 46–77% for Abu Roash C, D, E, F, and G members and the Bahariya Formation, respectively. Table 1 summarizes all calculated petrophysical parameters for all levels. Complementing these petrophysical trends, “Petrography and Microfacies” section uses thin-section analysis to unravel pore-scale controls on the reservoir quality quantified above.

**Table 1:** Summary of reservoir characteristics for the target formations, Abu Sennan Oil Field.

Formation	Reservoir interval	Well	Interval depths	Net pay thickness	Shale volume	Effective porosity	Oil saturation
				[m]	[%]	[%]	[%]
Abu Roash	AR/A	SWS-3	1459–1538.5	13	22	21	59
		SWS-5	1446–1506	–	–	–	–
		SWS-9	1527–1623	4	13	25	57
		SWS-18	1465–1605.5	68.50	27	17	67
	AR/B	SWS-3	1538.5–1605	0.38	8	12	63
		SWS-5	–	–	–	–	–
		SWS-9	–	–	–	–	–
		SWS-18	1605.5–1685.5	3	18	16	57
	AR/C	SWS-3	1605–1648.5	32.50	30	19	67
		SWS-5	1575–1619.5	2	29	19	62
		SWS-9	–	–	–	–	–
		SWS-18	1685.5–1732	1	35	29	52
	AR/D	SWS-3	1648.5–1731.5	9	6	19	77
		SWS-5	1619.5–1704	10.50	4	23	81
		SWS-9	1623–1665	10	2	21	90
		SWS-18	1732–1795.5	7.09	13	17	60
	AR/E	SWS-3	1731.5–1831.5	48.62	30	18	76
		SWS-5	1704–1800.5	–	–	–	–
		SWS-9	1665–1763	3.52	20	19	62
		SWS-18	1795.5–1877	3.41	25	24	69
	AR/F	SWS-3	1831.5–1851.5	16	13	19	84
		SWS-5	1800.5–1821	15.50	10	19	88
		SWS-9	1763–1781	16.48	9	20	90
		SWS-18	1877–1897	13.50	23	16	61
	AR/G	SWS-3	1851.5–1976	3	18	23	58
		SWS-5	1821–1941	5	11	22	66
		SWS-9	1781–1907	12.50	12	24	73
		SWS-18	1897–2020	5.50	14	23	65
Baharyia		SWS-3	1976–2234.5	5.50	21	26	46
		SWS-5	1941–2166.5	18.50	17	18	55
		SWS-9	1907–2162	52.50	27	15	77
		SWS-18	2020–2227	2.50	20	25	57

### Petrography and microfacies

#### Microfacies analysis of the Abu Roash “D” member (AR“D” m.)

The Abu Roash “D” member (AR“D” m.), located at the basal interval of the formation, exhibits a relatively uniform thickness of approximately 11 m. Microfacies analysis of this carbonate reservoir reveals distinct lithological and diagenetic characteristics across five intervals, as observed in the SWS-3 well. Below is a synthesized interpretation of the findings:

##### Mudstone to wackestone microfacies

**Fig. 9:**
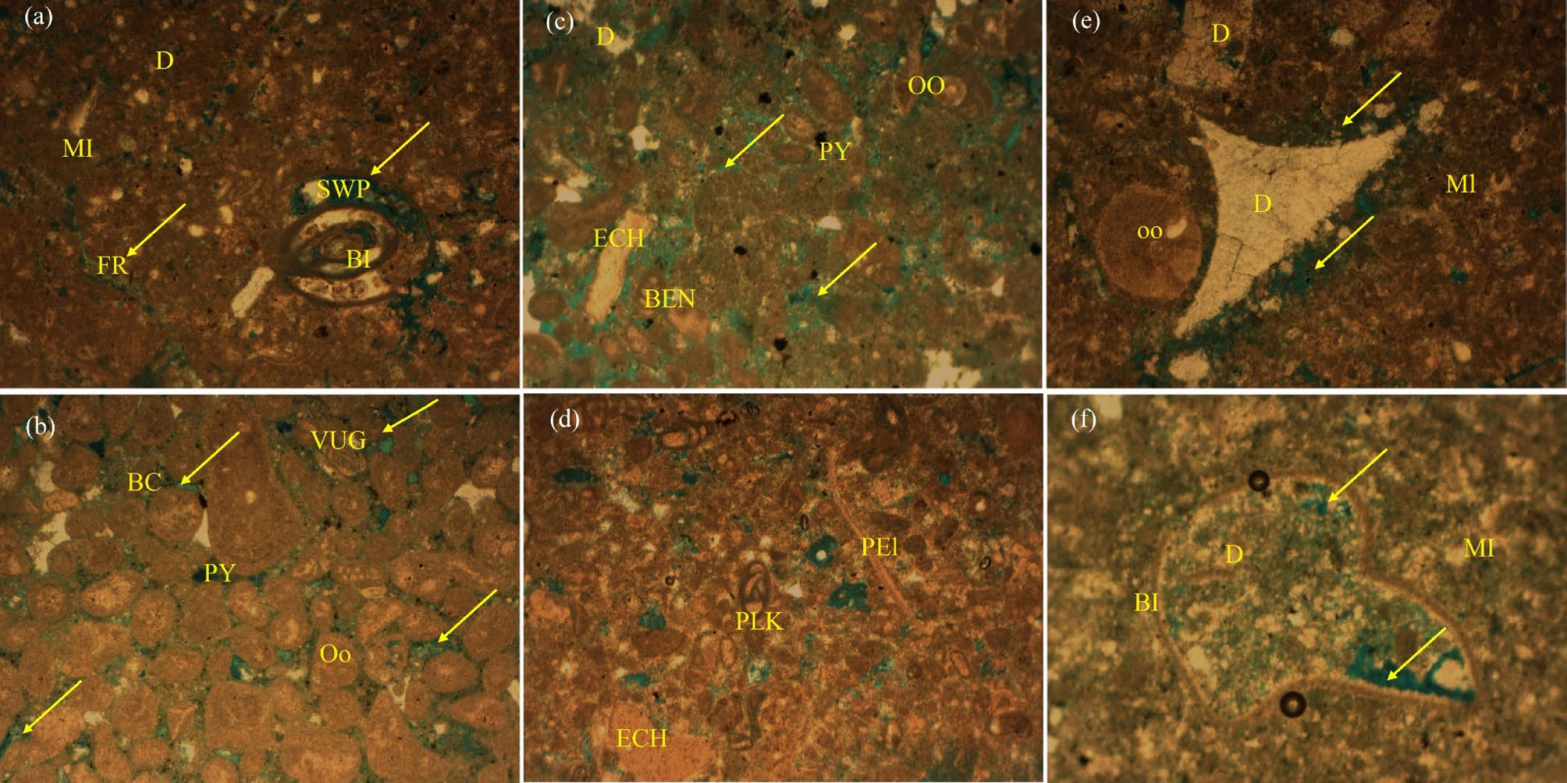
Thin-section photomicrographs (PPL) illustrating identified carbonate microfacies: (**a**) Mudstone to Wackestone, (**b**) Grainstone, (**c**) Bioclastic Grainstone, (**d**) Grainstone with Diverse Faunal Components, (**e**) Wackestone, (**f**) Wackestone Microfacies (Dunham classification).

This interval (1,788.33 m depth) is characterized by a micrite (MI)-dominated matrix with subordinate terrigenous clay and bioclast (BI) content, along with trace dolomite (D). Dissolution processes have moderately enhanced reservoir quality, generating porosity types including small vugs (VUG), intracrystalline pores (BC), secondary intraparticle pores (SWP), and fractures (FR) as documented in Fig. 9a. While porosity is limited, dissolution-derived features suggest potential fluid migration pathways (Fig. 9a).

##### Grainstone microfacies

At 1789.86 m depth, this microfacies comprises abundant ooids (OO) with minor terrigenous clay, dolomite, and pyrite (PY). The reservoir exhibits favorable characteristics due to well-developed vuggy and intracrystalline porosity, likely linked to high-energy depositional conditions and subsequent diagenetic modification (Fig. 9b).

##### Grainstone microfacies with biotic components

Sampled at 1790.11 m, this facies shares the ooid-rich framework of (b) but includes additional minor benthonic (BEN) and echinoid (ECH) fragments, alongside pyrite. The porosity network—dominated by vuggy and intracrystalline types—indicates active pore-preserving conditions, supported by minimal clay infiltration (Fig. 9c).

##### Grainstone microfacies with diverse faunal inclusions

Deeper at 1791.05 m, this unit displays a similar ooid-dominated fabric but incorporates pelecypod (PEL), planktonic (PLK), and echinoid fragments. Trace pyrite suggests reducing localized environments. The robust vuggy and intracrystalline porosity underscores strong reservoir potential, likely influenced by both primary deposition and secondary dissolution (Fig. 9d).

##### Wackestone microfacies

At 1792.44 m, this interval transitions to a micrite-rich matrix with minor terrigenous clay, ooids, dolomite, and pyrite. Reservoir quality is moderate, with porosity restricted to intracrystalline and secondary intraparticle types, reflecting lower energy deposition and limited diagenetic enhancement compared to grain-supported facies (Fig. 9e).

##### Wackestone microfacies

At 1793.14 m depth, this interval is dominated by micrite with minor terrigenous clay, ooids, dolomit, pyrite, and bioclasts. Reservoir quality is poor to moderate, with porosity limited to small vugs, intracrystalline, and secondary intraparticle pores. The micrite-rich matrix and limited dissolution restrict permeability, reflecting low-energy depositional conditions (Fig. 9f).

##### Mudstone to wackestone microfacies

**Fig. 10:**
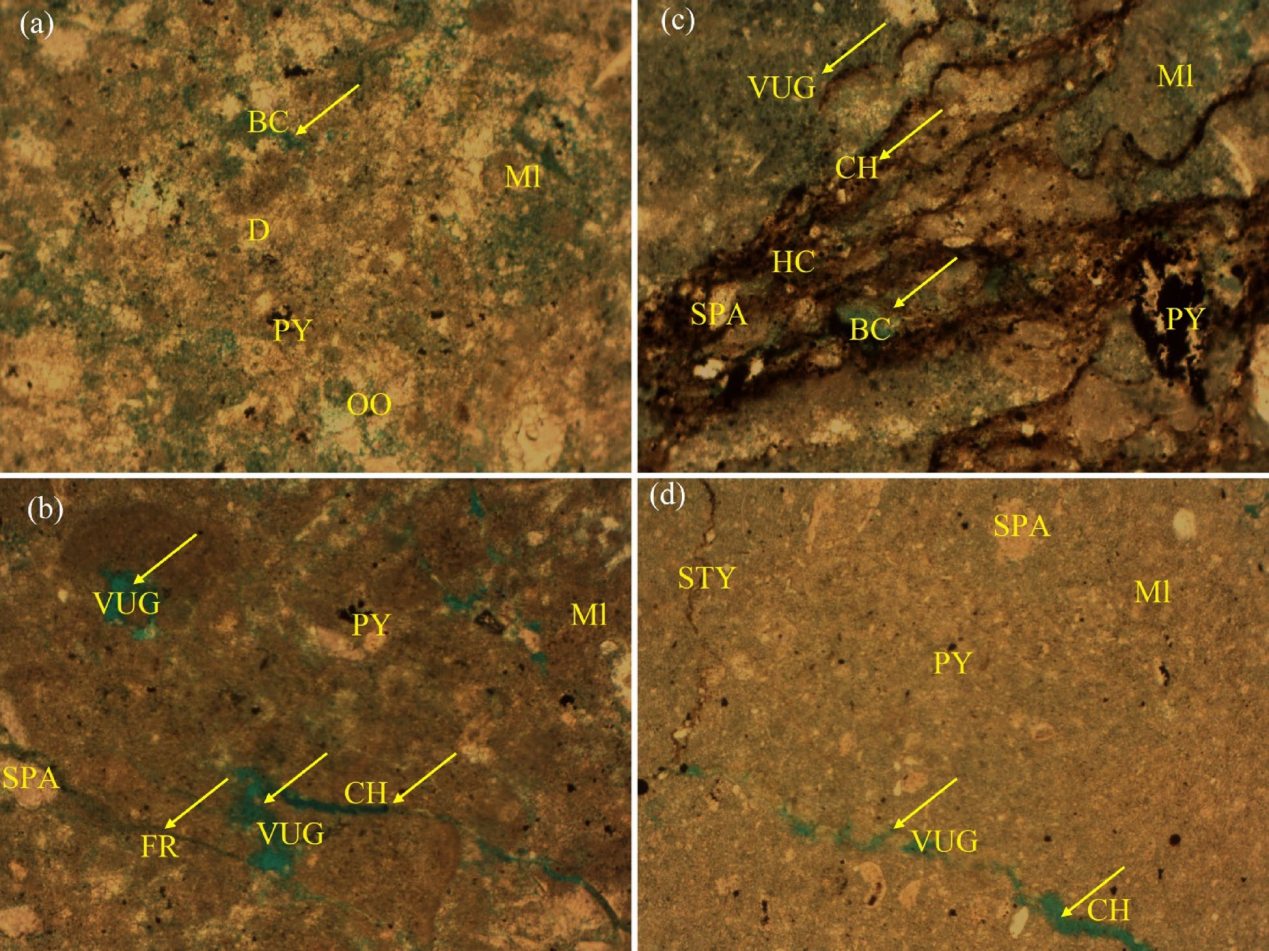
Thin-section photomicrographs (PPL) illustrating identified carbonate microfacies: (**a**) Mudstone to Wackestone, (**b**) Mudstone, (**c**) Hydrocarbon-Stained Mudstone, (**d**) Basal Mudstone Microfacies (Dunham classification).

Sampled at 1793.76 m, this unit comprises abundant micrite, rare ooids, minor terrigenous clay, and trace pyrite. Reservoir quality is poor, with porosity confined to sparse intracrystalline pores. The dominance of micrite and minimal grain support highlight deposition in a stagnant, restricted environment (Fig. 10a).

##### Mudstone microfacies

At 1795.99 m, these facies feature a micrite-rich matrix with minor terrigenous clay, sparite, and pyrite. Despite the presence of vugs, intracrystalline, secondary intraparticle, fracture, and channel (CH) porosity, reservoir quality remains poor due to pervasive micrite infilling and limited connectivity (Fig. 10b).

##### Hydrocarbon-stained mudstone microfacies

Notably, at 1796.96 m, this micrite-dominated interval contains minor sparite, terrigenous clay, and pyrite, alongside common residual hydrocarbons. Enhanced porosity—represented by vugs, intracrystalline, and channel pores suggests localized fluid migration pathways, resulting in unexpectedly high reservoir quality (Fig. 10c).

###### Basal mudstone microfacies

At 1797.51 m, the reservoir base consists of micrite with minor sparite, terrigenous clay, and pyrite. Porosity is very low, restricted to isolated vugs and stylolites, reflecting intense compaction and cementation in a shallow, restricted depositional setting (Fig. 10d).

#### Diagenetic controls on reservoir quality

The reservoir’s evolution was shaped by diagenetic processes acting over geological time, as documented by^59,60^.

##### Porosity-reducing factors

Physical Compaction: Mildly impacted reservoir quality, particularly in basal intervals (e.g., microfacies), where micrite-dominated fabrics were compressed into low-porosity strata.

Cementation: Authigenic minerals (e.g., iron oxides, calcite, argillaceous cements) occluded pore spaces. Calcite cementation, though limited, further reduced permeability.

##### Porosity-enhancing factors

Tectonic Fracturing: Cretaceous tectonic activity in the Abu Gharadiq Basin generated fractures, facilitating fluid migration and secondary porosity development.

Dissolution & Leaching: Acidic fluids, likely sourced from bounding faults, dissolved unstable grains, creating intracrystalline and vuggy porosity.

#### Depositional and structural insights

The AR“D” member’s vertical facies succession—from basal mudstones (restricted/shallow environments) to mid-section grainstones (shallow marine) and upper Wackestone—reflects sea-level oscillations within a subtidal carbonate factory.

Reservoir quality peaks in the mid-section (grainstone-dominated intervals (Fig. 9b–d) ), where dissolution and fracturing enhanced porosity to 17–24% (Table 1). Conversely, cementation and compaction degraded quality in micrite-rich units (Fig. 9a and Fig. 9e–f and 10a–d). Proximity to structural highs and faults was critical, as these zones allowed acidic fluids to percolate, amplifying dissolution and fracture porosity.

This study integrates microfacies heterogeneity, diagenetic overprints, and structural controls to unravel the AR“D” reservoir’s complexity. The middle intervals, influenced by high-energy deposition and tectonic-dissolution interplay, represent optimal targets for hydrocarbon exploration. In contrast, micrite-rich basal and upper units, though locally influenced by hydrocarbon migration, remain secondary due to diagenetic degradation. These findings underscore the importance of multi-scale analysis in carbonate reservoir characterization.

### Seismic interpretation

**Fig. 11:**
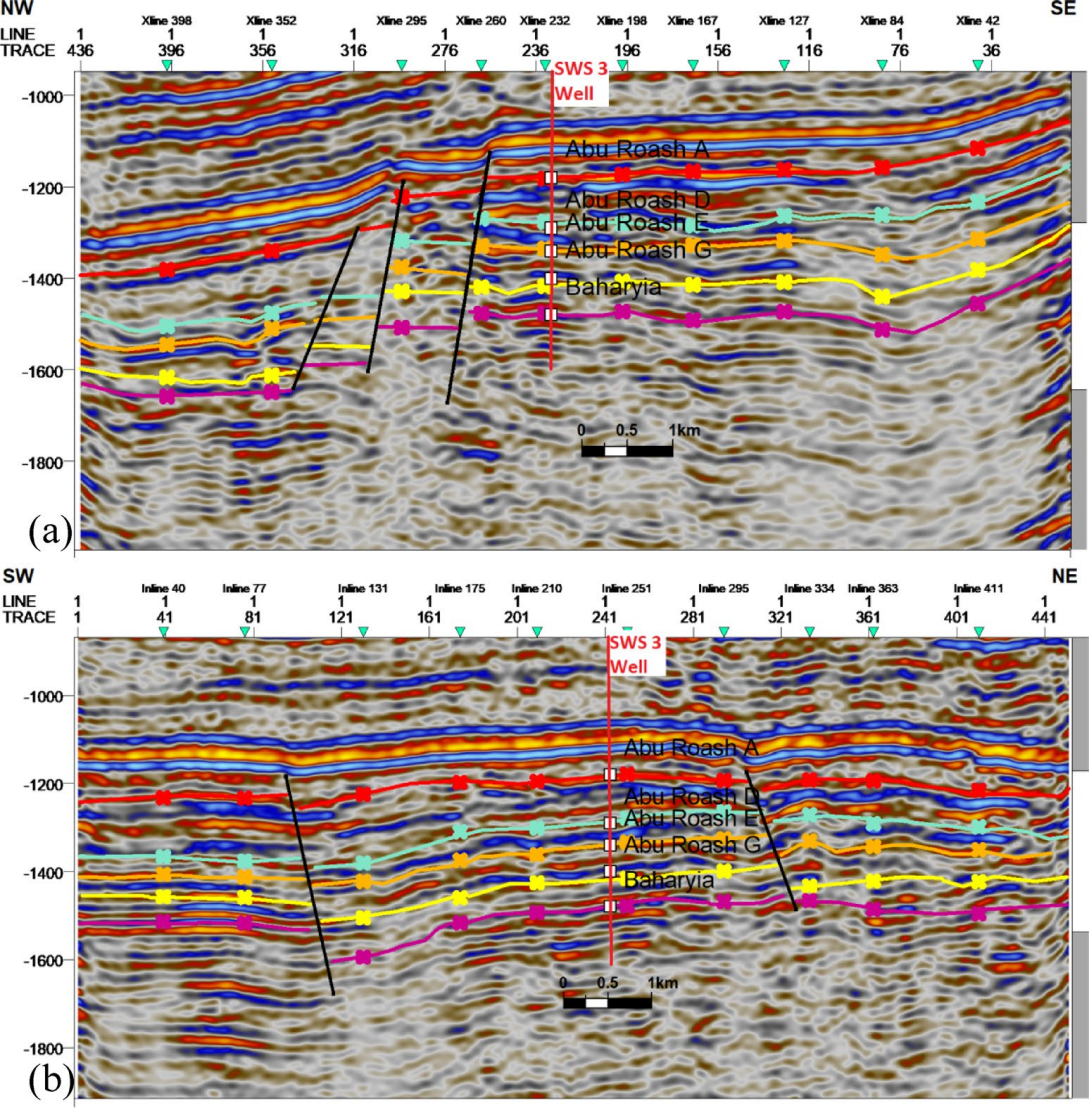
Seismic sections demonstrating well-to-seismic tie calibration and primary seismic interpretation.

**Fig. 12:**
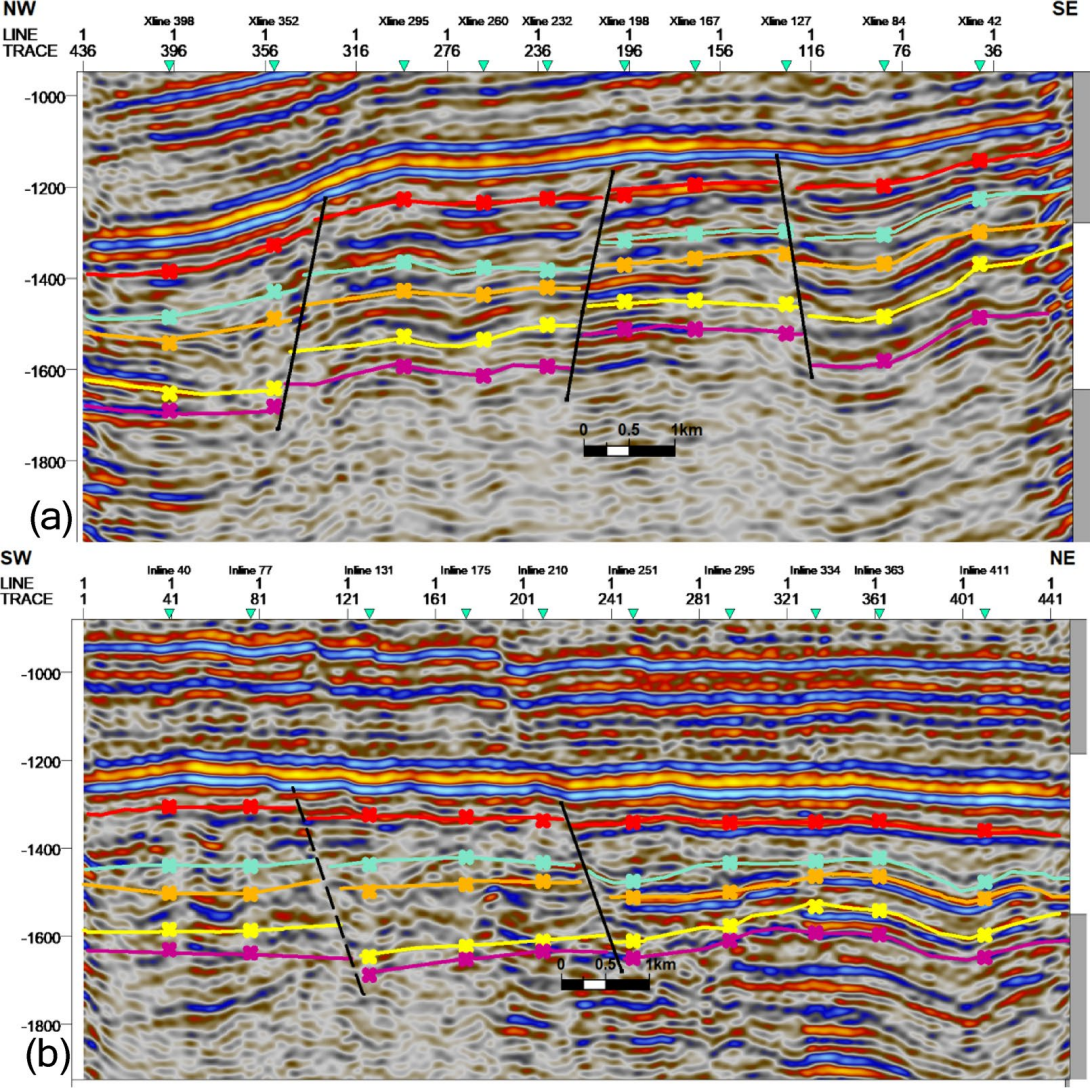
Interpreted seismic sections displaying picked horizons and identified fault systems.

**Fig. 13:**
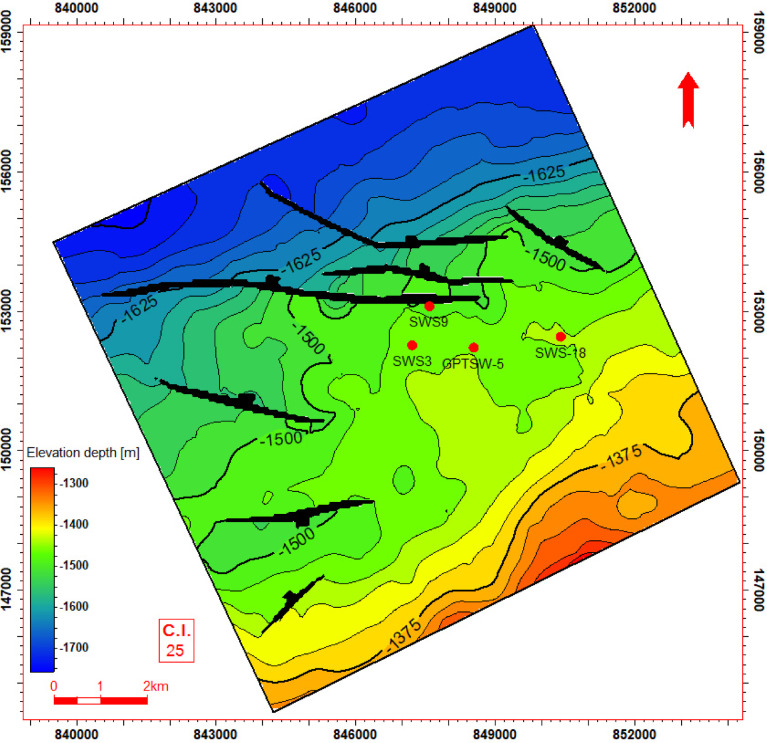
Depth structure contour map (meters TVDSS) for the Top Abu Roash C horizon, illustrating fault geometry and structural configuration.

**Fig. 14:**
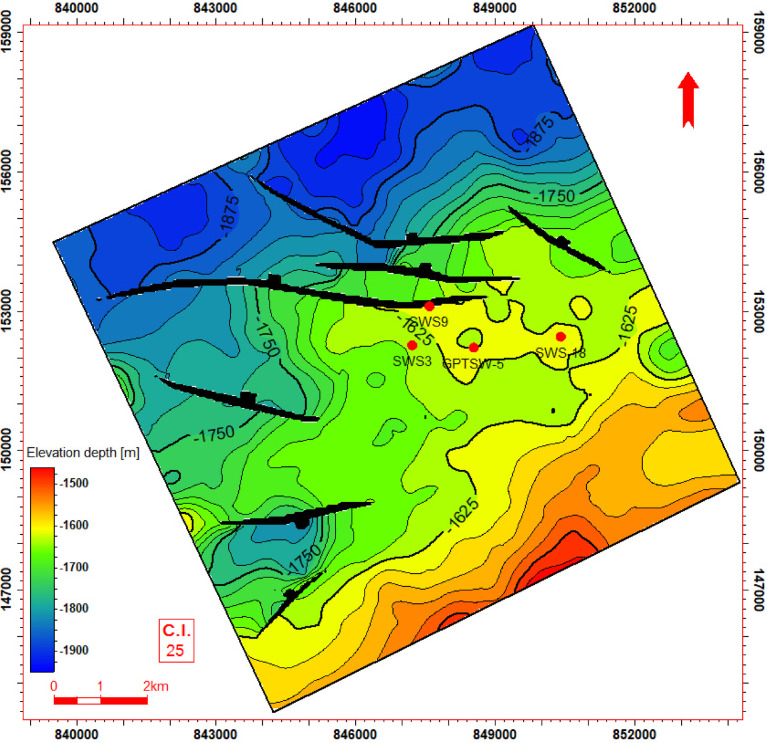
Depth structure contour map (meters TVDSS) for the Top Abu Roash D horizon, illustrating fault geometry and structural configuration.

**Fig. 15:**
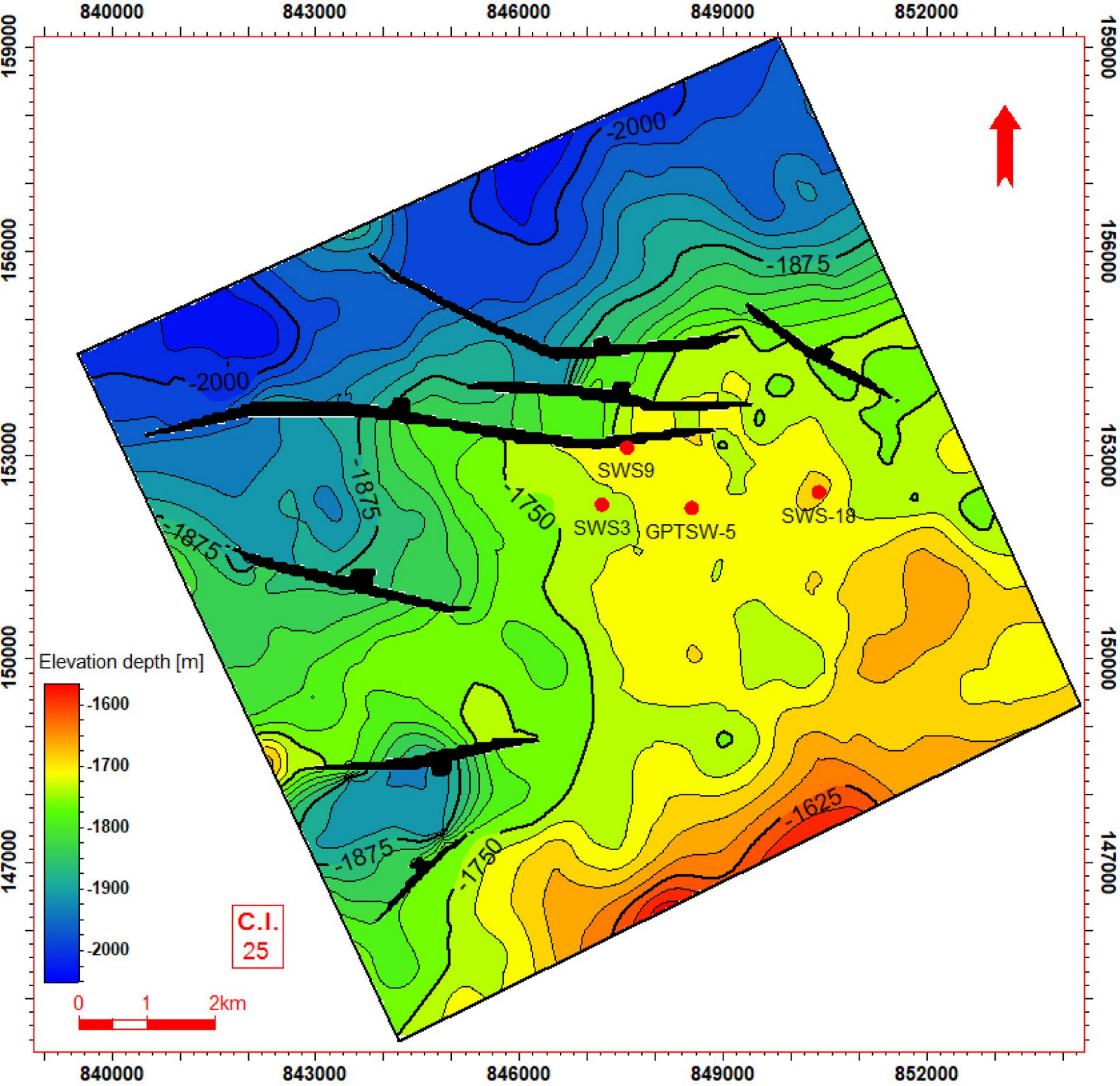
Depth structure contour map (meters TVDSS) for the Top Abu Roash E horizon, illustrating fault geometry and structural configuration.

**Fig. 16:**
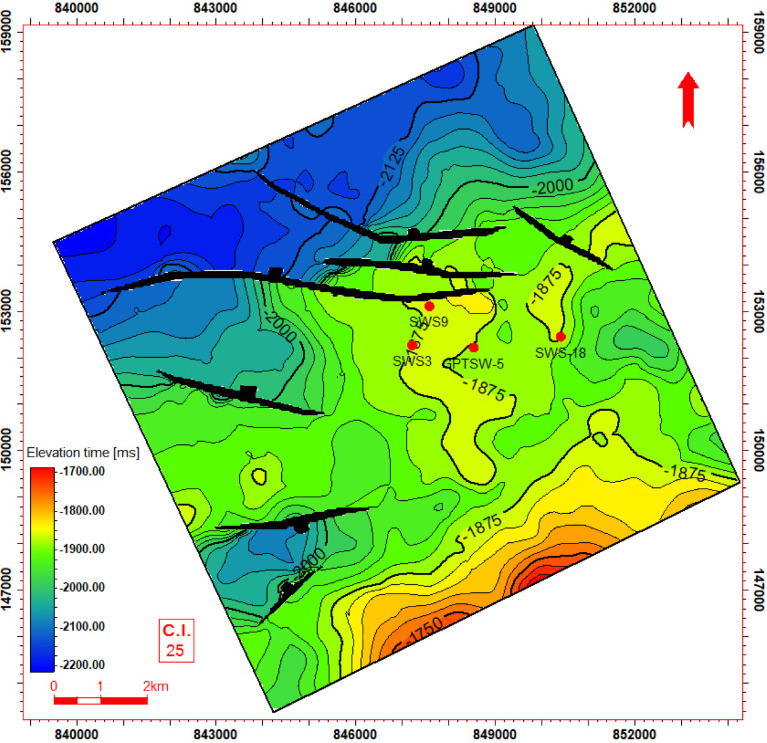
Depth structure contour map (meters TVDSS) for the Top Abu Roash G horizon, illustrating fault geometry and structural configuration.

**Fig. 17:**
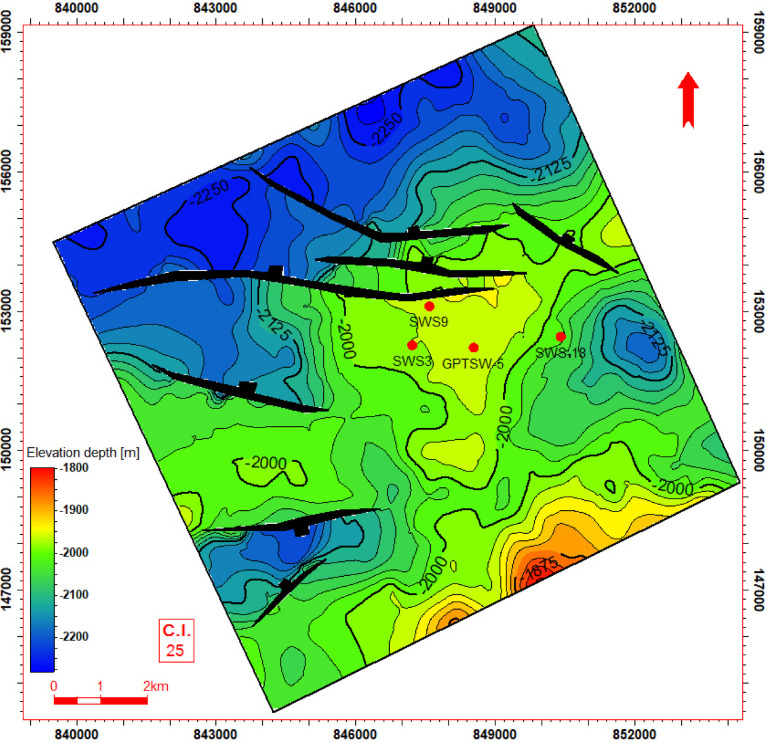
Depth structure contour map (meters TVDSS) for the Top Bahariya Formation horizon, illustrating fault geometry and structural configuration.

Seismic-to-well tie, a foundational step in seismic interpretation, was performed through the integration of check-shot data from wells across the study area to establish accurate time-depth relationships (Fig. 11). Figures 11 and 12 present four interpreted seismic profiles, delineating key stratigraphic horizons (Abu Roash A, D, E, G members, and Bahariya Formation) and fault architectures. Figure 10 highlights the seismic-well tie and structural interpretation along two orthogonal orientations: (a) a NW–SE trending inline (251) and (b) a NE-SW trending cross line (232). The seismic data reveals a network of normal faults organized into step-fault systems, characterized by tilted fault blocks, horst-graben structures, and variable fault throws, indicative of an extensional tectonic regime. Figure 12 further details the fault geometry and kinematics. Profile 12a (NW–SE inline 131) delineates three normal faults, including one fault with a SE-dipping hanging wall and two faults exhibiting NW-dipping hanging walls. Profile 11b (NE–SW cross line 352) illustrates two normal faults with NE-dipping hanging walls. These fault systems, interpreted within the seismic volume, demonstrate compartmentalization of the reservoir units and offer analyses of the paleostress field and basin evolution. Depth-converted seismic structure maps of the Abu Roash A, D, E, and G members, alongside the Bahariya Formation (Figs. 13, 14, 15, 16 and 17), delineate three dominant fault trends linked to regional tectonic phases. These trends include (1) a NW–SE orientation (Gulf of Suez trend), (2) an E–W alignment (Mediterranean trend), and (3) a NNW–SSE direction, interpreted as a reactivated Paleozoic–Triassic structural fabric^61^. The integration of these maps facilitated the construction of a 3D structural model for the Abu Sennan area, capturing the interplay of multi-phase tectonism across the Abu Roash and Bahariya Formations.

The maps highlight fault geometries, displacement patterns, and structural culminations critical to reservoir compartmentalization. Kinematic analysis of the NW–SE and E–W trends suggests Cenozoic extensional regimes, while the NNW–SSE trend reflects inherited basement-involved deformation, consistent with regional paleostress field evolution. This multi-trend framework underscores the complex superposition of tectonic events that shaped basin architecture and influenced hydrocarbon migration pathways.

The fault systems mapped in “Seismic Interpretation” section directly constrain the 3D structural model described in “Reservoir Modeling”, section where compartmentalization impacts fluid saturation distributions (Fig. 21).

### Reservoir modeling

**Fig. 18:**
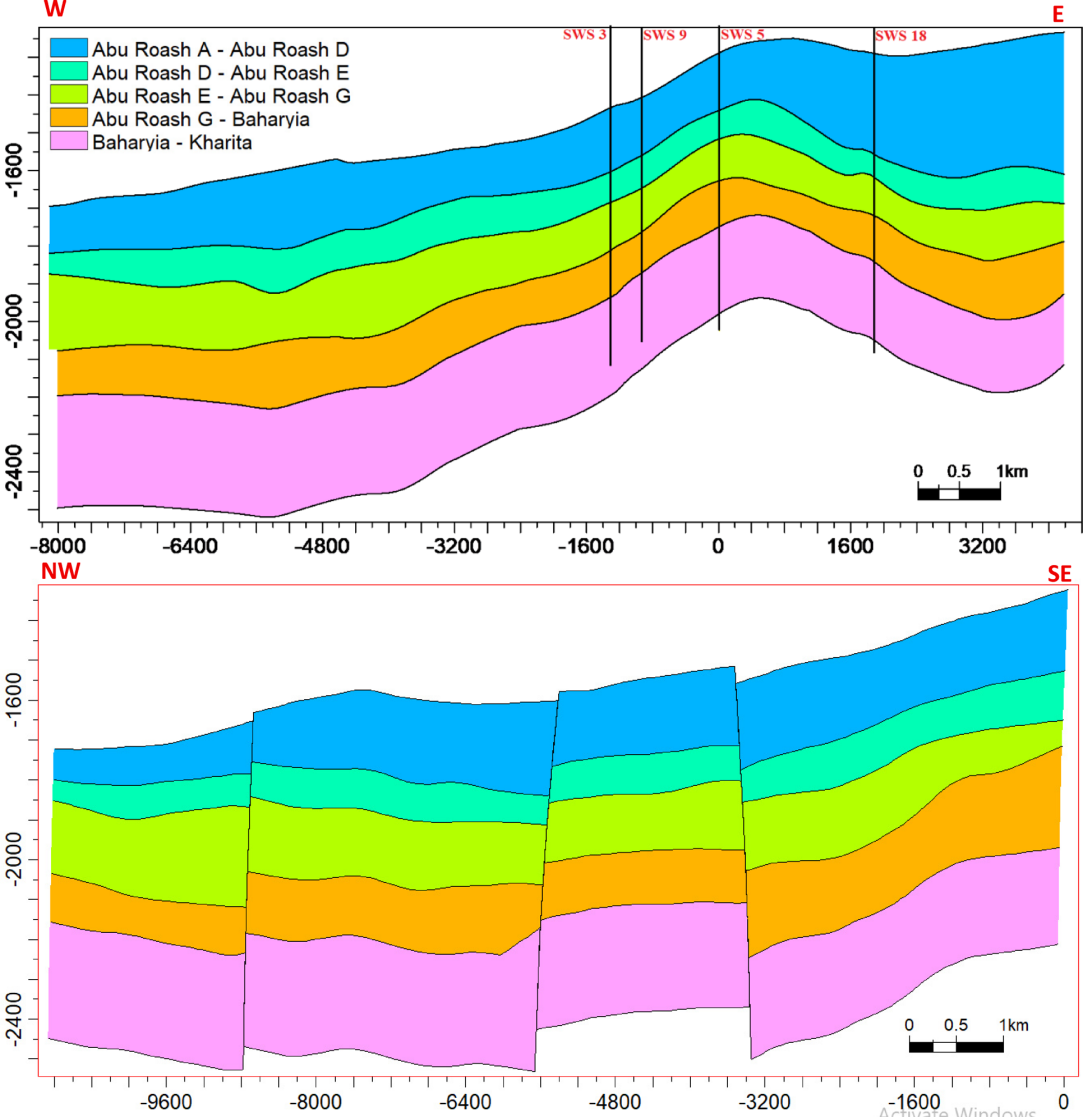
3D geological model cross-sections along lines B (upper panel) and A (lower panel), depicting interpreted fault networks and their spatial relationships.

**Fig. 19:**
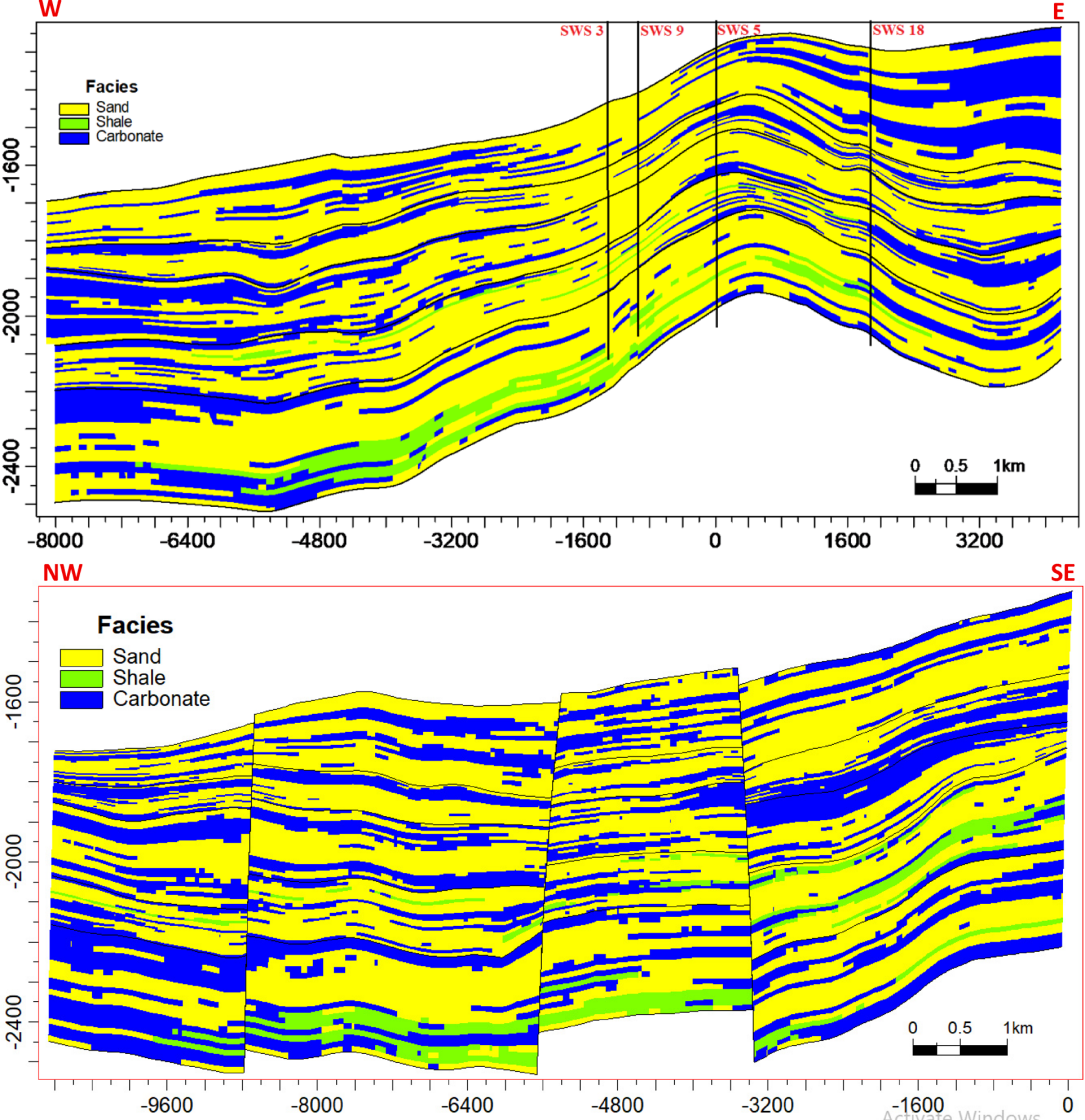
3D facies model cross-sections along lines B (upper panel) and A (lower panel), illustrating the spatial distribution of depositional facies.

**Fig. 20:**
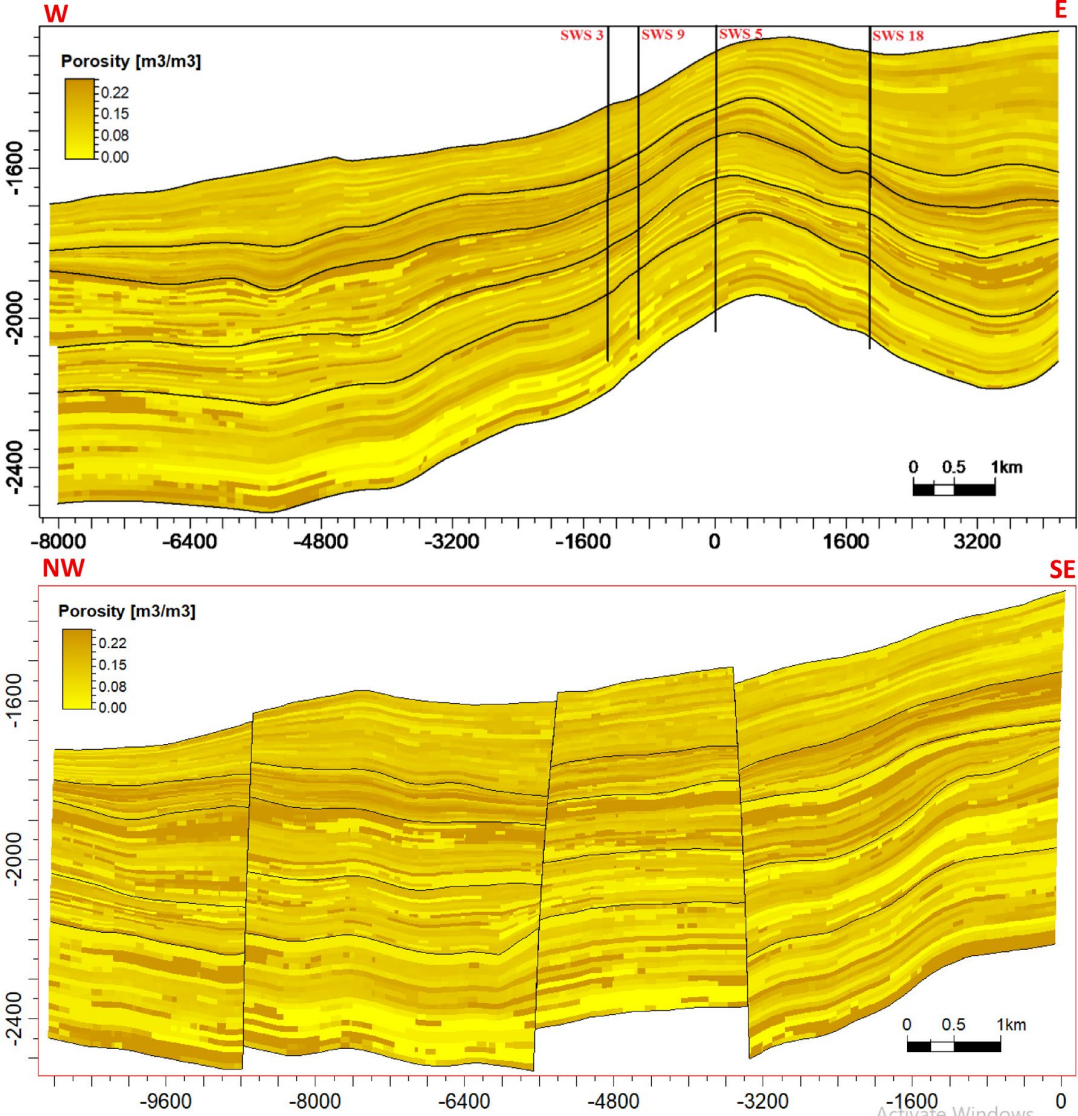
3D effective porosity model cross-sections along lines B (upper panel) and A (lower panel), depicting the spatial distribution of effective porosity (v/v).

**Fig. 21:**
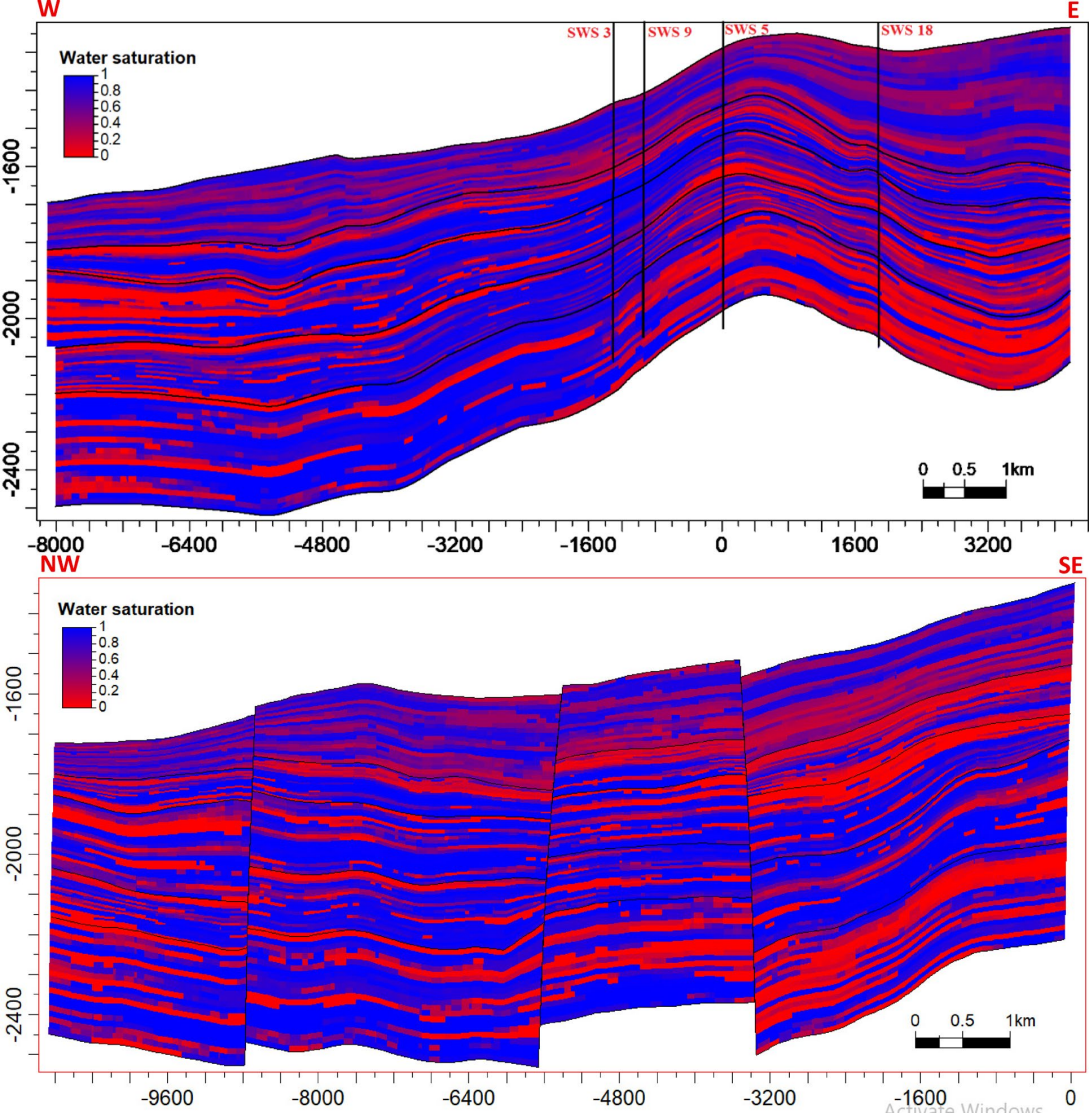
3D hydrocarbon saturation model cross-sections along lines B (upper panel) and A (lower panel), illustrating the spatial distribution of hydrocarbon saturation (v/v).

The reservoir modeling workflow advanced with stratigraphic horizon interpretation, utilizing depth-converted structural maps of the Abu Roash A–G members and Bahariya Formation. Cell thickness calibration optimized the vertical discretization of the 3D grid, resolving lithological heterogeneity at sub-seismic scales. A representative cross-section of the structural model (Fig. 18) reveals NE–SW-oriented normal fault systems, including step faults and a central horst structure. This fault framework compartmentalizes the reservoir, with the horst acting as a high-relief structural trap, creating up-dip closures conducive to hydrocarbon entrapment. Lithofacies analysis identified three dominant lithotypes—sandstone, limestone, and dolomite—within the Abu Roash C member to Bahariya Formation interval (Fig. 19). Based on core-log-seismic integration, we spatially distributed these facies, reflecting transitions from shallow-marine to fluvio-deltaic depositional environments. Subsequent petrophysical modeling (Figs. 20, 21) quantifies effective porosity and hydrocarbon saturation across the Abu Roash A–G members and Bahariya Formation, highlighting reservoir quality variations tied to diagenetic overprints and facies-controlled porosity–permeability relationships.

### Hydrocarbon volumetrics

A primary objective of the 3D reservoir model construction is the accurate quantification of the Stock Tank Oil Initially In Place (STOIIP). The finalized model enables robust volumetric calculation, providing the foundation for reserves assessment and field development planning. The resulting STOIIP distribution across key reservoir units is presented in Table 2.

**Table 2:** Stock Tank Oil Initially In Place (STOIIP) distribution by reservoir zone, calculated from the 3D reservoir models.

Zones	STOIIP (in oil) [*10^6 sm3]
Abu Roash A	23,095
Abu Roash D	10,729
Abu Roash E	15,015
Abu Roash G	15,644
Baharyia	29,847

The Baharyia formation contains the largest in-place volume (29.8 × 10^6^sm^3^), confirming its status as the primary reservoir target. The significant heterogeneity in resource distribution across the Abu Roash intervals (A, D, E, G) is quantitatively captured, with Abu Roash A holding the second-largest volume (23.1 × 10⁶ sm^3^). These results validate the geological and petrophysical integrity of the model and provide the critical input for subsequent dynamic simulation and recovery factor analysis.

## Discussion

The Bahariya Formation comprises sandstone with shale intercalations, consistent with what is stated by^27^**.** The Bahariya Formation is a source rock, as reported by^62^, and a promising reservoir rock, as stated by^62^. The Abu Roash Formation, overlaying the Bahariya Formation, comprises limestone and sand with shale intercalations as observed from the lithological identification cross plots (Figs. 2, 3, 4, 5, 6) and thin sections (Figs. 9, 10). It is subdivided into seven members, from the A member at the top to the G member at the bottom. This comprehensive study recognized and mapped the structure-affected Abu Sennan area. The seismic interpretation (Figs. 11, 12) revealed various structural regimes from that of other authors^63,64^. This interpretation showed that no reverse faults exist in this portion of the study area. The first trend is the NW–SE trend (Gulf of Suez trend), the second is the east–west trend of the Mediterranean, and the third is the NNW–SSE trend representing the tectonic movement of the Paleozoic to Triassic, according to^61^, as illustrated from the depth-structure-contour maps of the Abu Roash members and the Bahariya Formation This study further evaluated the area’s petrophysical parameters of the Abu Roash and Bahariya formations. Unlike^21^, this work studied all Abu Roash and Bahariya Formations’ members. Besides, this study included building 3D static reservoir models.

The integration of well log interpretation, petrographic analysis, seismic interpretation, and reservoir modeling provides a robust framework to unravel the controls on reservoir heterogeneity and hydrocarbon potential in the Abu Roash and Bahariya Formations. This multidisciplinary approach bridges pore-scale diagenetic processes to basin-scale structural evolution, offering critical insights into hydrocarbon exploration in analogous carbonate systems.

### Reservoir quality and diagenetic controls

The Abu Roash “D” member exemplifies the interplay between depositional facies and diagenesis in shaping reservoir quality. High-energy grainstone microfacies (Figs. 9b–d) exhibit significant porosity (17–24%, Table 1, Fig. 20) and oil saturation (60–90%, Table 1; Fig. 21) due to primary interparticle porosity and dissolution-enhanced vuggy/intracrystalline pores. These facies, deposited in shallow marine settings, were further upgraded by Cretaceous tectonic fracturing and acidic fluid migration along bounding faults, which amplified secondary porosity. In contrast, micrite-dominated mudstone-wackestone facies, indicative of low-energy restricted environments, suffered porosity reduction (≤ 15%, Table 1; Fig. 20) from pervasive micrite infilling and calcite cementation. Notably, the hydrocarbon-stained mudstone challenges conventional facies-quality paradigms, suggesting localized migration pathways exploited fractures and channels despite a micritic matrix.

These findings align with global carbonate reservoir studies^59,60^, where dissolution and fracturing counterbalance compaction/cementation. However, the AR“D” member’s unique diagenetic overprint—particularly the role of fault-controlled acidic fluids—highlights the importance of structural plumbing systems in enhancing otherwise marginal facies.

### Structural compartmentalization and hydrocarbon entrapment

Seismic interpretation reveals a complex structural architecture dominated by NW–SE (Gulf of Suez) and E–W (Mediterranean) fault trends, superimposed on inherited NNW–SSE Paleozoic fabrics. The NE–SW-oriented horst structure (Figs. 13, 14, 15, 16, 17) acts as a high-relief trap, with up-dip closures formed by antithetic faults creating compartmentalized reservoirs. This structural framework directly controls hydrocarbon distribution: high oil saturations (60–90%, Table 1; Fig. 21) in Abu Roash D and F members correlate with fault-bounded horst blocks, where fractures enhanced permeability. Conversely, the Bahariya Formation’s variable saturation (46–77%, Table 1; Fig. 21) reflects poorer connectivity in micrite-rich, fault-isolated compartments.

The multi-phase tectonic evolution—Cenozoic extension reactivating Mesozoic-Paleozoic structures—mirrors regional basin dynamics (e.g.,^37^). This superposition created migration conduits along fault planes while simultaneously compartmentalizing reservoirs, underscoring the dual role of faults as both enhancers and barriers in carbonate systems.

### Depositional heterogeneity and petrophysical uncertainty

Lithofacies transitions from fluvio-deltaic (Bahariya) to shallow marine (Abu Roash) are encoded in the 3D reservoir model (Fig. 19). High porosity (19–29%) and saturation (52–90%) in Abu Roash C–G members align with grainstone dominance, while the Bahariya’s lower porosity (15–26%) reflects sandstone-dolomite intercalations with detrital clay. However, the model’s vertical discretization, though optimized via cell thickness calibration, remains limited by seismic resolution, particularly in thin-bedded units (e.g., AR“D” micrite layers). This uncertainty underscores the need for stochastic modeling to capture sub-seismic heterogeneity.

### Prospect identification

The integration of interpreted 2D seismic data, petrophysical analysis, and structural mapping culminated in the construction of detailed 3D static reservoir models for the key Abu Roash (C, D, E, and G members) and Bahariya formations within the Abu Sennan Field. These models, capturing both the complex structural architecture and the spatially distributed reservoir properties (facies, effective porosity, hydrocarbon saturation), served as the primary tool for identifying and evaluating new exploration targets.

Systematic analysis of the depth-structure maps derived from seismic interpretation revealed several structural closures (fault-dependent closures) within the study area that had not been previously targeted. Crucially, the 3D static models allowed these structural features to be evaluated in the context of the overlying seal integrity, predicted reservoir facies distribution, effective porosity, and hydrocarbon saturation patterns derived from well control and seismic data where applicable.

**Fig. 22:**
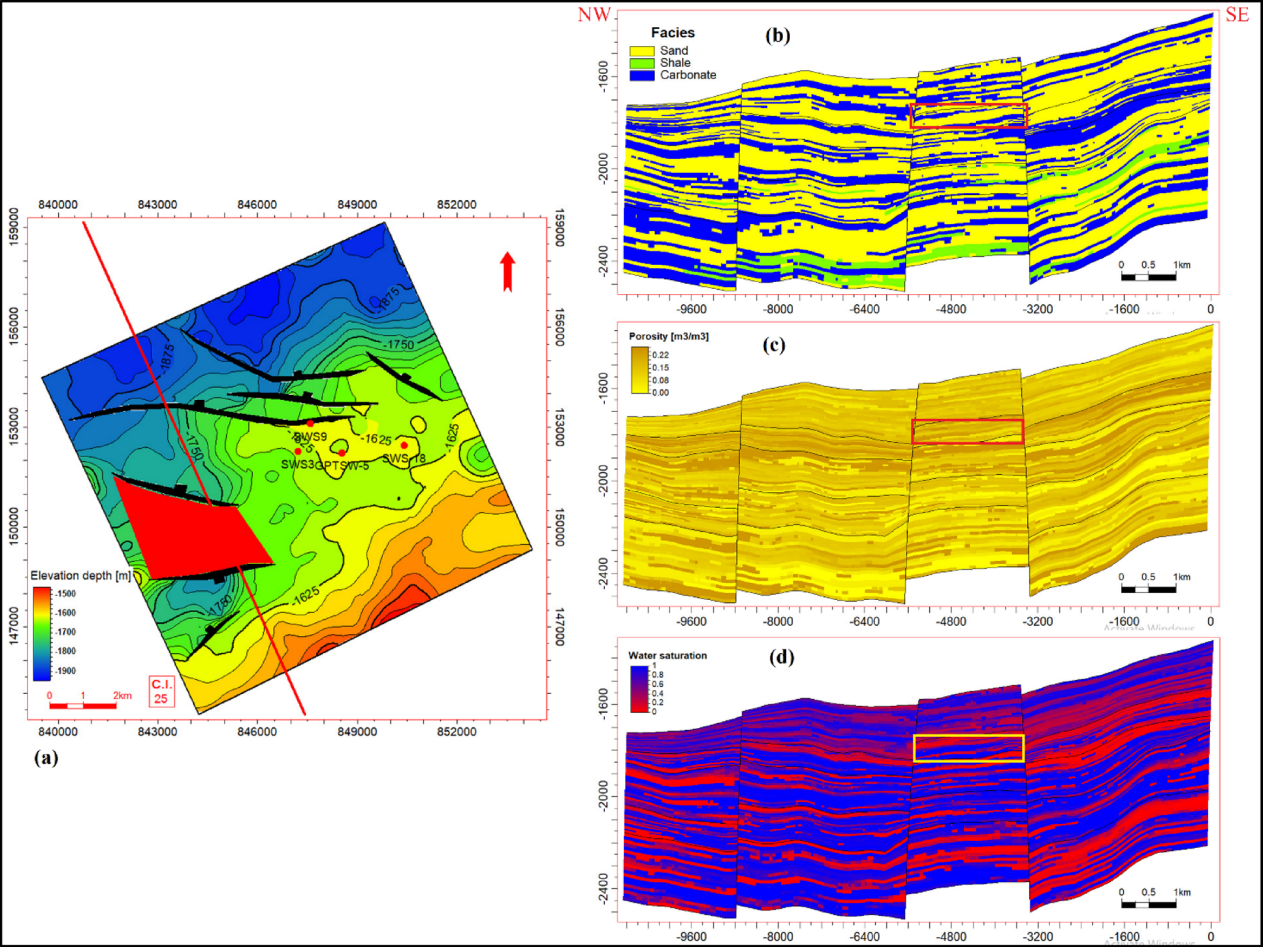
Prospect AR-D-01 in Abu Roash ‘D’. (**a**) Depth structure map of Top AR-D showing fault-bounded closure (red polygon). (**b**) NE-SW section of 3D facies model (sandstone: yellow; carbonate: blue; shale: green) highlighting prospective area (red rectangle). (**c**) Effective porosity (Φ, %) section showing high-porosity zone. (**d**) Hydrocarbon saturation (Sₕ, fraction) section confirming high-saturation zone within structural/porous sweet spot, validating prospect potential.

Prospect AR-D-01, Fig. 22a, represents a high-potential structural closure identified through systematic analysis of the seismic-derived structural map of top AR” D” and reservoir property distributions from our static models (Fig. 22b, c, and d). It demonstrates robust potential with (1) fault-bounded closure (Fig. 22a), (2) 22–24% effective porosity (Fig. 22c), and (3) > 70% hydrocarbon saturation (Fig. 22d) in AR“D” grainstones (Fig. 22b).

#### Limitations and future research directions

While this study provides a comprehensive reservoir characterization of the Abu Roash and Bahariya formations, several limitations warrant acknowledgment:

6 Data Resolution Constraints: Our 3D static models integrate 2D seismic data and well logs from 5 wells, but seismic resolution limits thin-bed detection (< 15–20 m vertical resolution). Higher-resolution 3D seismic or advanced attribute analysis could better resolve sub-seismic heterogeneities.

7 Core Data Coverage: Petrographic analysis was restricted to the AR“D” member in a single well (SWS-3). Expanding core sampling across all Abu Roash members and additional wells would strengthen diagenetic trend predictions.

8 Dynamic Validation: The static models (Figs. 18, 19, 20, 21) lack calibration to production data or dynamic simulation. Future work should incorporate reservoir performance history to test model predictability.

These limitations inspire critical next steps:

1. Acquisition of 3D seismic surveys to enhance fault/facies imaging.
2. Multi-well core-based diagenetic studies to quantify cementation/compaction trends basin-wide.
3. Dynamic model coupling to assess volumetric uncertainties and optimize development plans for identified prospects

## Conclusions

This comprehensive study of the Abu Roash and Bahariya formations elucidates the synergistic interplay of depositional, diagenetic, and tectonic processes governing reservoir heterogeneity and hydrocarbon potential in the Abu Sennan area. Key conclusions are as follows:

1. *Reservoir quality controls*
  The Abu Roash “D” member exhibits the highest reservoir potential among studied intervals, with grainstone microfacies, 17–24% porosity, and 60–90% oil saturation (Table 1, “Petrography and Microfacies” section) benefiting from high-energy deposition and fault-enhanced dissolution. Conversely, micrite-rich mudstone-wackestone facies exhibit diminished quality due to compaction and cementation, except where localized fracturing or hydrocarbon migration pathways create unexpected sweet spots.
2. *Structural architecture*
  Seismic interpretation reveals a multi-phase tectonic history, with NW–SE (Cenozoic) and NNW–SSE (Paleozoic) fault trends compartmentalizing the reservoir into horst-graben traps. The central horst structure (Fig. 18) acts as a high-relief trap, with fault-bounded compartments correlating to elevated hydrocarbon saturations (up to 90% in Abu Roash D and F, Table 1, Fig. 21).
3. *Depositional-diagenetic synergy*
  Reservoir quality peaks where shallow-marine grainstones intersect fault zones, enabling acidic fluid influx and secondary porosity development. Fluvio-deltaic Bahariya facies, while porous (15–26%) Table 1, Fig. 20, suffer from detrital clay infiltration, reducing permeability.
4. *Exploration implications*
  The middle Abu Roash interval (D), particularly near fault intersections, represents prime targets for drilling. Bypassed pay potential exists in fractured micrites and hydrocarbon-stained mudstones, warranting advanced stimulation strategies. High shale content in Abu Roash C and Bahariya necessitates tailored drilling practices to mitigate formation damage.

## Data Availability

Data sets generated during the current study are available from the corresponding author on reasonable request, but restrictions apply to the availability of these data.
